# PFAS Molecules: A Major Concern for the Human Health and the Environment

**DOI:** 10.3390/toxics10020044

**Published:** 2022-01-18

**Authors:** Emiliano Panieri, Katarina Baralic, Danijela Djukic-Cosic, Aleksandra Buha Djordjevic, Luciano Saso

**Affiliations:** 1 Department of Physiology and Pharmacology “Vittorio Erspamer”, Sapienza University of Rome, 00185 Rome, Italy; luciano.saso@uniroma1.it; 2 Department of Toxicology “Akademik Danilo Soldatović”, Faculty of Pharmacy, University of Belgrade, 11211 Belgrade, Serbia; katarinab@pharmacy.bg.ac.rs (K.B.); Danijela.djukic.cosic@pharmacy.bg.ac.rs (D.D.-C.); aleksandra.buha@pharmacy.bg.ac.rs (A.B.D.)

**Keywords:** PFAS, PFOA, PFOS, human health, ecosystem, remediation technologies

## Abstract

Per- and polyfluoroalkyl substances (PFAS) are a group of over 4700 heterogeneous compounds with amphipathic properties and exceptional stability to chemical and thermal degradation. The unique properties of PFAS compounds has been exploited for almost 60 years and has largely contributed to their wide applicability over a vast range of industrial, professional and non-professional uses. However, increasing evidence indicate that these compounds represent also a serious concern for both wildlife and human health as a result of their ubiquitous distribution, their extreme persistence and their bioaccumulative potential. In light of the adverse effects that have been already documented in biota and human populations or that might occur in absence of prompt interventions, the competent authorities in matter of health and environment protection, the industries as well as scientists are cooperating to identify the most appropriate regulatory measures, substitution plans and remediation technologies to mitigate PFAS impacts. In this review, starting from PFAS chemistry, uses and environmental fate, we summarize the current knowledge on PFAS occurrence in different environmental media and their effects on living organisms, with a particular emphasis on humans. Also, we describe present and provisional legislative measures in the European Union framework strategy to regulate PFAS manufacture, import and use as well as some of the most promising treatment technologies designed to remediate PFAS contamination in different environmental compartments.

## 1. Introduction

Per-and polyfluoroalkyl substances (PFAS) constitute an heterogeneous group of fluorinated synthetic compounds characterized by the presence of at least one perfluorinated methyl group (−CF_3_) or a perfluorinated methylene group (−CF_2_−), a variable number of carbon atoms, fluorination degree and presence of other chemical groups. PFAS are almost ubiquitous into the environment, mainly due to their wide dispersive use and applicability in a vast number of industrial sectors and consumer products [1,2]. Increasing concern for human health and wildlife ecology derives from the thermal and chemical stability of PFAS molecules and the multiple routes through which humans and biota can be exposed during their lifetime [3,4,5,6,7]. Of note, while the PFAS family has rapidly expanded into an impressive number of more than 4700 different substances including both the “legacy PFAS” (i.e., PFOS, PFOA) and the “emerging PFAS” (e.g., GenX) [8,9] producers, decision makers as well as researchers try to gain insights on their impact and to find the most appropriate measures to mitigate the potential risks associated with their exposure. Common features of PFAS are represented by their chemical stability which causes environmental persistence [10], their high mobility which confers them a long-range transport potential [11] causing their pervasive spreading even into remote regions (e.g., the Arctic’s or Antarctic’s) [12,13,14] and their tendency to bioaccumulate and biomagnify in biota through the contamination of the food chains [15,16,17,18,19]. The presence of some PFAS has been reported in the blood [20,21], milk [22,23], urine [24] tissues [25,26,27] and organs [28,29,30,31,32] of different human populations living in developed countries and has been associated to a number of adverse health effects. Similarly, relevant concentrations of PFAS have been detected in the air [33,34], groundwater [35,36], freshwater [17,37], marinewater [38,39], drinking water [40,41] and soil [42,43,44] potentially causing ecotoxic effects in the aquatic and terrestrial ecosystems at the trophic levels of primary producers, primary consumers and secondary consumers [45,46]. An additional layer of complexity is given by the coexistence of different mixtures of PFAS substances and other contaminants in the environmental media, for which quantitative risk assessment analysis and toxicologic/ecotoxicologic information is still scarce if not absent [47,48]. Therefore, it is of utmost importance to rapidly fill the gap of scientific knowledge and to develop not only the adequate analytical methods to detect PFAS contamination and the high-throughput approaches to predict PFAS toxicity but also effective strategies of legislative regulation, remediation and therapeutic interventions to mitigate potential effects on humans and biota. On the other hand, it is advisable that the industry and the governments would cooperate to progressively promote a more sustainable innovation and a shift towards less hazardous substances by issuing also appropriate guidelines or regulatory measures to regulate and monitor PFAS environmental releases.

## 2. PFAS Properties, Uses, Sources and Their Distribution into the Environmental Compartments

### 2.1. PFAS Classification

Although it might be quite surprising, a universally accepted definition of PFAS is still lacking. This is due to the fact that PFAS definition is constantly evolving based on the scope, application and criteria adopted by different studies conducted on this broad category of substances [49]. From an historical perspective, a first classification was proposed around 2011 by Buck and coworkers in a seminal paper wherein PFAS were defined as “the highly fluorinated aliphatic substances that contain 1 or more C atoms on which all the H substituents have been replaced by F atoms, in such a manner that they contain the perfluoroalkyl moiety C_n_F_2n+1_–” [1]. Some years later, in 2018, the Organisation for Economic Co-operation and Development (OECD), reported the existence of several PFAS molecules that, despite having fully fluorinated carbon atoms, were devoid of the –CF_3_ group and thus did not meet the previous definition of Buck et al. [50]. In the attempt to reconcile these discrepancies, a quite recent report by the OECD has proposed a broader definition of PFAS as: ”fluorinated substances that contain at least one fully fluorinated methyl or methylene carbon atom (without any H/Cl/Br/I atom attached to it), i.e., with a few noted exceptions (represented by a carbon atom instead having H/Cl/Br/I atoms attached), any chemical with at least a perfluorinated methyl group (−CF_3_) or a perfluorinated methylene group (−CF_2_−) is a PFAS” [51].

**Figure 1:**
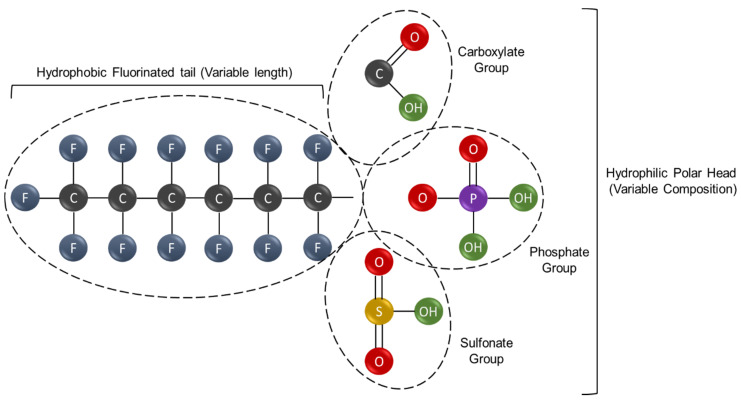
Overview of the general structure of non-polymeric, perfluorinated PFAS substances. Non-polymeric PFAS are compounds of variable composition and physicochemical properties that however share two common features. These are represented by the hydrophobic tail, composed by a variable number of carbon atoms at different degree of fluorination, and the hydrophilic head, which contains polar groups. The specific combination of these chemical determinants, namely the carbon chain length, the type of functional groups and the number of fluoride atoms, generates an enormous number of different PFAS molecules with ample downstream applicability. Some of the most common polar groups, are shown.

**Figure 2:**
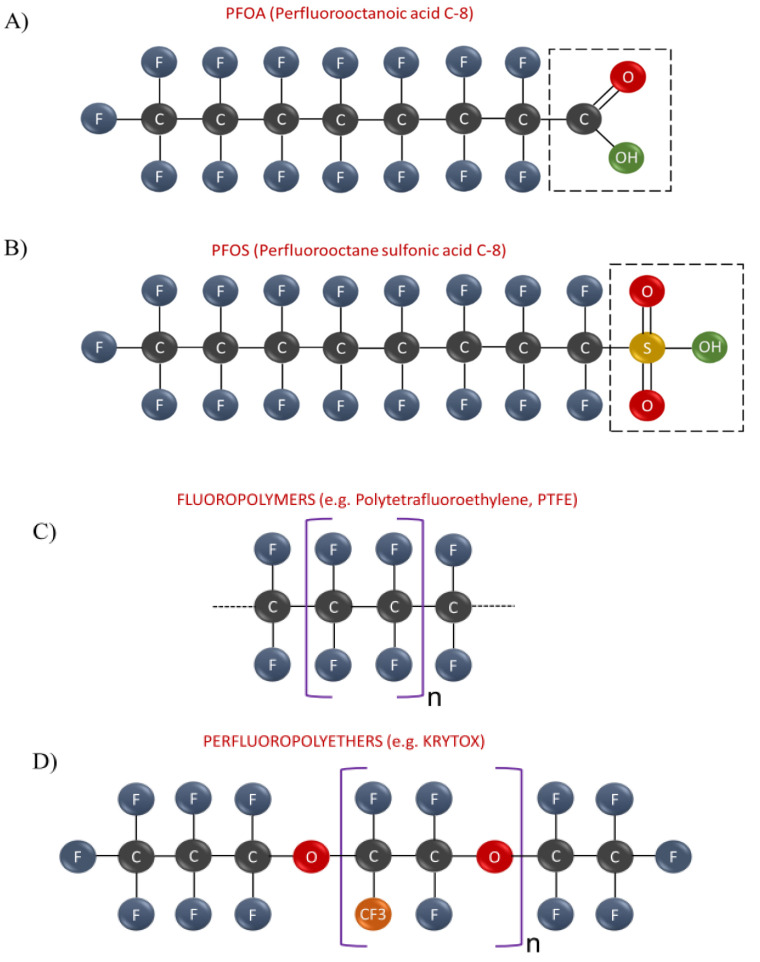
Examples of non-polymeric and polymeric PFAS substances. (**A**,**B**) PFOA and PFOS, two well-known non-polymeric PFAS are shown. Both these compounds possess a relatively long tail containing eight fluorinated carbon atoms, but differ in the chemical composition of the polar head group, which is a carboxylic acid for PFOA and a sulfonic acid for PFOS. Under specific pH environmental conditions, these functional groups can dissociate into the respective anion forms (i.e., carboxylate and sulfonate). (**C**) Exemplary structure of Polytetrafluoroethylene (PTFE), also known as Teflon, a polymeric PFAS belonging to the subgroup of fluoropolymers. This type of compound is constituted by a moiety of (CF2-CF2)_n_ atoms which is repeated up to thousands of times; (**D**) Exemplary structure of a lubrificant also known as Krytox, which is a polymeric PFAS belonging to the subgroup of perfluoropolyethers. In this case the (CF[CF3]−CF2−O)_n_ moiety is repeated between 10–60 times.

For what concerns PFAS classification, the length of the fluorinated carbon chain, which ranges between C4-C17, is often used as a main discriminant and a good predictor of physicochemical properties, bioaccumulation, protein-binding as well as environmental fate distribution [52,53,54,55,56]. According to the well-recognized PFAS classification system edited by Buck and coworkers [1], PFAS can be grouped into two broad categories: non-polymeric and polymeric molecules. Non-polymeric PFAS can be further subdivided into two groups represented by perfluoroalkyl and polyfluoralkyl substances. The former includes molecules wherein the hydrophobic carbon chain is totally fluorinated with the exception of the terminal end, which hosts a polar functional group such as carboxylate (COO−), sulfonate (SO_3_−) or phosphate (OPO_3_^−^) which confers hydrophilicity [10,52] (see Figure 1). Of note, the perfluoroalkyl PFAS can be further subdivided into heterogeneous subgroups (see Table 1) among which the perfluoroalkyl acids (PFAAs) include some of the most well-known and extensively studied molecules such as perfluorooctane sulfonate (PFOS) and perfluorooctanoic acid (PFOA) (see Figure 2A,B). By contrast, the group of polyfluoroalkyl PFAS encompasses molecules wherein at least one (but not all) the carbon atoms are partially fluorinated and bound to oxygen or hydrogen atoms (e.g., 6:2 FTOH). On the other hand polymeric PFAS include: (a) fluoropolymers, substances wherein most if not all hydrogen atoms of the carbon chain are replaced by fluoride atoms (e.g., PTFE, PVDF); (b) side-chain fluorinated polymers, substances constituted by non-fluorinated carbon chains of variable composition and poly/perfluoroalkylic side chains (e.g., fluorinated acrylate polymers); (c) perfluoropolyethers (PFPEs), substances wherein the main backbone contains oxygen atoms and fluoride atoms directly bound to the carbon chain (e.g., PFPE-BP) (see Figure 2C,D and also Table 1).

### 2.2. PFAS Uses

The exceptional strength of the C-F bond confers very high thermal and chemical stability to PFAS molecules while the contemporary presence of hydrophobic and hydrophilic properties along with the variability in the carbon chain length and chemical composition generates an enormous range of different molecules with useful physicochemical properties. For this reason, since 1940’s, PFAS substances have been successfully employed in a vast number of industrial or consumer’s products covering more than 100 sectors of use. In this respect, some of the most common applications include pesticide formulation, firefighting foams, cosmetics, aerospace, aviation, automotive, textiles coating, oil production, medical products, food processing, building and construction, energy, paper and packaging, cables and wiring, electronic and semiconductors (see Table 2) [2,58,59]. A list of the PFAS presented in this review, with their names and acronyms is found in Table 3.

### 2.3. Sources of PFAS Emissions

**Figure 3:**
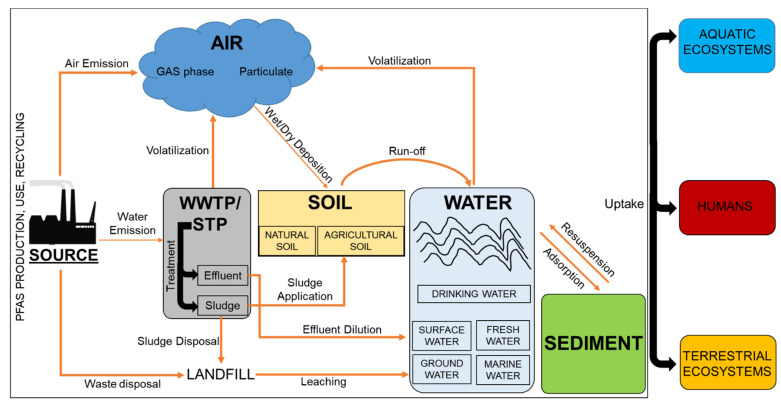
Schematic illustration of the PFAS environmental distribution and exposure routes for humans and biota. The environmental distribution of PFAS substances involves multiple dispersion routes and exposure pathways that link the sources to the final receptors, represented by humans and wildlife. Industrial manufacture processes, industrial uses and recycling activities, represent primary sources of PFAS emissions. Other indirect sources are the landfilling or the application of contaminated sludge to agriculture land. Volatilization, deposition, leaching and run off processes regulate the redistribution of PFAS between air, soil, water and sediment compartments. Collectively, these pathways contribute to the short-term and long-term exposure of aquatic ecosystems, terrestrial ecosystems and humans to PFAS substances, that can also enter into the food chain through bioaccumulation and indirect human exposure via the ingestion of contaminated food sources.

Local and diffuse sources of PFAS emission into air are represented by the industrial production of fluoropolymers, building construction, food packaging, textiles, medical devices, paints, or the professional use of firefighting foams, printing inks and paints [58,59,60,61]. Additional releases derive from the use and disposal of consumers products such as cosmetics, personal-care products, textiles, household products as well as materials for food storage and processing [62,63]. Major sources of PFAS pollution derive from the industrial and municipal waste-water treatment plants (WWTPs), which can directly contribute to PFAS release into the atmosphere and freshwater systems through the discharge of contaminated effluents or indirectly promote PFAS dispersion into soil through the spreading of contaminated sewage sludge, recycled wastewater and biosolids for agriculture uses [64,65,66,67]. Other relevant sources of atmospheric emissions are represented by the industrial plants used for recycling and incineration of PFAS-containing products or the landfilling of wastes which under specific conditions can leach into soils, ultimately entering into groundwater [36,67,68,69]. A schematic representation of PFAS emission sources is shown in Figure 3. Recent reports estimate that the number of potential sites hosting installations emitting in some quantity PFAS, is in the order of 100,000 or more in the sole EU, a number which might further increase in the next future [70]. Based on these considerations, it is not surprising that PFAS have become pervasive environmental pollutants impacting on the ecosystems and human health through the contamination of multiple routes of human exposure via the environment including the atmosphere, the drinking water, the cereals, fruits, vegetables, milk and other food sources [71,72,73,74,75].

### 2.4. Environmental Fate of PFAS

By virtue of their physicochemical properties and their widespread use, PFAS have been detected in almost every region of the globe, in different environmental media, living organisms and human populations [70,76,77,78,79]. It is well recognized that PFAS are characterized by extreme resistance to thermal, chemical or biotic degradation which causes environmental persistence and poses serious concerns to the ecosystems and human health [10]. However, some polyfluorinated PFAS can undergo partial degradation under certain environmental conditions and this can result in the formation of other PFAS substances with even greater impact than their precursors, as in the case of PFAAs [1]. One very well-known example is represented by the atmospheric oxidation of fluorotelomer alcohols which leads to the generation of the corresponding polyfluorinated aldehyde, further transformed into PFCAs substances [80]. It is also known that PFOS and PFOA can be present as impurities and be directly released from industrial sources or disposal of consumers products [81,82] but also derive from biotic/abiotic degradation or biotransformation processes of longer-chain PFAS molecules [83,84] and other precursors such as 8:2 FTOH [85,86]. Moreover, PFAS are amphiphilic and can bioaccumulate within the adipose tissue or the bloodstream of living organisms, while their high mobility renders their environmental distribution ubiquitous due to leaching into groundwater, run-off into streams and oceans, wind dispersion through dust particulates and wet/dry deposition into soils [79,87,88,89,90]. A schematic illustration of the PFAS environmental distribution and fate is shown in Figure 3. In the following sections, the main targets of PFAS contamination, including humans and the relevant environmental compartments, will be described more in detail.

#### 2.4.1. PFAS Occurrence in the Atmosphere

While the vast majority of PFAS have a low tendency to evaporate due to generally low vapor pressure, low Henry constant and high boiling point, other neutral species, such as perfluorooctane sulfonates (FOSAs) or sulfamidoethanols (FOSEs) as well as flurotelomer alcohols (FTOHs) or acrylates (FTACs), are instead volatile or semivolative and can partition into the atmosphere [91,92] (see Figure 3). For example, in a study aimed to characterize the exposure and risks of vulnerable subpopulations in Germany, variable levels of FTOHs, FTACs, Et/MeFOSA, and Et/MeFOSE were detected in the indoor air from schools and residences air samples contributing to non-oral intake exposure [93]. Similarly, by assessing PFAS occurrence in the atmosphere gradient across the Atlantic and the Southern Ocean, 12 different neutral PFAS were found at a total concentration ranging from 2.8 to 68.8 pg/m^3^, with a clear prevalence of FTOHs [94]. Consistently, measurements in the atmosphere over the northern South China Sea, led to the identification of four distinct types of PFAS molecules belonging to the FTOHs (which were the most abundant), FTAs FOSAs, and FASEs subgroups, at an average total concentration of 54.5 pg/m^3^ [95]. Of note, even poorly volatile PFAS can have a high sorption potential and affinity for the organic matter (high log Koc, high log Koa), which facilitates their transport into the air after adsorption to the atmospheric particulate matter. For instance, a study was conducted on distinct locations from three different countries, Japan, China and India, to analyze the seasonal and local changes in PFAS adsorbed to air particulate ranging from 0.1 to 10 µm (PM_10_-PM_0,1_). Here, ultrafine particles in the nanosize range (i.e., PM_0,1_) were found to be major contributors to the mass fraction of PFCAs, revealing a prominent abundance of PFOA, PFNA and PFDA substances while the highest PFOS mass fraction was associated with larger size particles [96] in agreement with a more recent investigation in North Carolina [33]. Importantly, a similar mechanism might contribute to the environmental dispersion of alternative PFAS with shorter carbon chain length (C < 8). This is evidenced by a recent study wherein, among the others (i.e., PFOS, PFOA, PFHxA), the hexafluoropropylene oxide dimer acid (HFPO-DA), was detected at a concentration ranging from <0.086 to 21.5 pg/m^3^ in the PM samples collected from Asian cities [97]. Similarly, Yu and coworkers made use of a cryogenic air sampler to collect atmospheric particulate and gas phase, for the subsequent identification of PFAS molecules through nontarget analysis. Here, 38 classes and 117 homologues of PFAS were identified revealing a prevalent occurrence of many PFSAs, some chlorinated perfluoropolyether carboxylic acids and twelve different hydrosubstituted perfluoroalkyl carboxylates (H-PFCAs) in the PM. The authors concluded that novel chlorinated polyether PFAS substances currently used as alternatives to legacy PFAS, require future investigation to elucidate their environmental impact in the ecosystems [98]. As already mentioned, the atmospheric dispersion of PFAS substances has profound consequences in their subsequent transport towards multiple environmental media and receptors since wet/dry deposition mechanisms can account for a significant portion of PFAS entrance into the aquatic and terrestrial ecosystems [42,99,100,101,102]. Taken together these data demonstrate that the atmospheric dispersion of PFAS is a relevant pathway through which these compounds can diffuse towards human and potentially other biotic receptors residing far distant from the source of emission.

#### 2.4.2. PFAS Occurrence in the Aquatic Systems

It is well known that some PFAS subgroups such as ionic PFAAs with short carbon chain length are highly water-soluble and can partition at the water-air interface [103,104,105], while longer chain PFAs (C ≥ 6) and PFCAs (C ≥ 8) tend to be distributed into the water-sediment fraction and biota [106,107,108] (see Figure 3). For this reason, significant amounts of these compounds are constantly detected in a large number of water bodies and aquifers. Waters have a crucial impact in the ecosystems, since they cover two-thirds of our planet hosting a huge number of living organisms, they serve as a source of drinking water for humans and they are the final receptor of pollutants from all the other compartments. Thus, in the following paragraphs we will briefly describe some of the most significant studies assessing PFAS occurrence and effects in water systems.

##### PFAS Occurrence in Freshwater and Sediments

Freshwater accounts for a small fraction of the overall Earth water content but includes a variety of ecosystems such as lakes, rivers, wetlands, streams and groundwater aquifers which are of vital importance for the mitigation of climate variability and the circulation of energy, nutrients and organisms between other environmental media [109]. At the same time, freshwater resources are essential for humans, providing supply water for several manufacture processes, drinking, agriculture, energy and food production [109]. As evidenced by many reports, PFAS have been detected in various freshwater sites worldwide (see Figure 3). For example, a study on Lake Victoria and its source rivers led to the identification of significant amounts of PFOA and PFOS derived from the emission of generic point sources such as domestic and industrial waste. Here, PFOA and PFOS levels in the river waters ranged from 0.4–96.4 ng/L and 0.4–13.23 ng/L, respectively while lower amounts were detected in the lake waters, with values of 0.4–11.65 ng/L for PFOA and 0.4–2.53 ng/L for PFOS [110]. From the analysis of three sites affected by industrial and mining pollution along the Vaal River in South Africa, comparable levels of PFAS were reported by Groffen and coworkers, suggesting also a potential risk for human health through the intake of contaminated fish [111]. In other sites such as the Hubei province in China, variable concentrations of 12 different PFAS substances have been detected in the Qing River, with values peaking during summer in a range from 39.44 to 207.59 ng/L [112]. Another investigation was conducted on the river–lake system along the Yangtze River in Jiangxi Province to reveal the presence of 11 PFAS species in the surface waters presumably of WWTPs origin. The authors found high levels of total PFAS in the surface waters of the Nanchang City urban area (ranging from 146.2 to 586.2 ng/L) and the Jiujiang section of the Yangtze River (ranging from 46.2 to 157.6 ng/L) with a marked prevalence of PFBS and PFOA [113]. Importantly, severe water contamination can occur when AFFFs are used during training or emergencies as also confirmed by a quite recent study assessing the PFAS concentration in the sediment, lake and pond water located in the surrounding area of a firefighting training facility in Sweden. Here, the authors found the presence of 9 PFAS in the water lake with a total average concentration of 1700 ng/L while in the sediment 6 different PFAS were detected at an overall concentration in the range of <1.0 ng/g dw and 76 ng/g dw. The composition profile revealed a dominance of PFSAs (in particular PFHxS, PFOS and PFBS) and PFCAs (in particular PFHxA) subcategories in the water lake samples and a relative contribution of PFOS and PFHxS of 71% and 23% of the overall sediments PFAS concentration [106]. Seasonal variations in the levels of 13 different PFAS were also reported in a study analyzing water and sediment status of the Ebro Delta and surrounding coastal areas in Spain. Here, PFOA was found to be the predominant compound in freshwaters with an average concentration of 1.6, 0.97 and 0.87 ng/L for autumn, winter and spring, respectively. On the other hand, sediments were more enriched in PFOS, with levels ranging from 1.02 to 22.6 ng/g dw and characterized by a trend of progressive decrease from summer to winter [114]. Another area of interest has been the Port Philip Bay located around the city of Melbourne in Australia. By analyzing the levels and occurrence of common PFAS in surface waters from seven creeks and estuaries, Allinson et al. identified eighteen PFAS substances, including PFBS, PFOA, PFBA, PFHxS and PFOS at concentrations up to 7.0 ng/L, 8.5 ng/L, 11 ng/L, 42 ng/L and 75 ng/L, respectively. Based on these data, the authors concluded that the presence of PFAS in urban and rural freshwaters might occur also in countries with relatively low population density [115]. Another relevant site is the Rhone River, which is considered the main freshwater and sediment supplier of the Mediterranean Sea [116] and has been the focus of recent studies. An interesting investigation from Mourier et al. reconstructed the temporal variation of PFAS levels in sediments collected in a section of the Rhone River along the city of Lyon (France) from 1984 to 2013. Here, the authors observed three distinct patterns with an initial increase of the total PFAS concentration from 2 ng/g dw prior to 1984, up to 51.4 ng/g dw in 1994 and a subsequent decrease to around 10 ng/g dw, which remained stable since the late 2000s. The data revealed the presence of multiple sources of contamination and a local discharge of both short-chain (PFHxA) and long-chain (PFNA, PFUnDA) PFCAs as well as a progressive shift from odd to even perfluorinated long-chain PFCAs substances, with a relatively stable proportion of PFSAs, thus indicating a change in the PFAS manufacturing [117]. A subsequent study monitored the presence of four PFAS in the Rhone River from 2017 to 2018. Here, the authors found high levels of total PFAS (range of 13–200 ng/L), high amounts of PFHxA (range of 8–193 ng/L) and PFOS concentrations exceeding the annual average European Environmental Quality Standards (EQS) in more than 80% of the cases, concluding that the Rhone River might represent an important source of PFAS to the Mediterranean Sea [118]. In another study Codling et al. analyzed the contamination status of the surface sediments and cores from the Great Lakes Erie and Ontario as well as Lake St. Clair in North America. Here, 20 different PFAS substances were identified at a total mean concentration ranging from 15.6 to 19 ng/g dw revealing PFHxA and PFOS as the most frequently detected compound and PFBA as the most abundant (ranging from 14.2 to 26.2 ng/g dm)[119]. Also, a recent work has assessed the concentration of PFAS in both water and sediment cores from the Lake Sänksjön in Sweden, previously impacted by the release of PFAS-containing AFFFs. Here, the authors identified eight different PFAS in the lake water, and thirteen PFAS in the sediment samples, ranging from 95–100 ng/L and 3–61 ng/g dw, respectively. In both the cases, the PFAS composition was markedly dominated by PFOS and PFHxS [120]. In another report, by using liquid chromatography tandem mass spectrometry (LC-MS/MS), Guo et al. analyzed the concentration of PFSAs PFCAs, PFPIAs and PFPAs from sediment cores of distinct lakes in the Great Lakes Region, revealing that the highest contamination level occurred in the Ontario lake (sum of PFAS equal to 13.1 ng/g), where PFOS contributed over 80% of the total content [121]. Taken together these data indicate that PFAS spreading into freshwater systems can be considered ubiquitous and largely influenced by seasonal variations and by the presence of multiple non-point and point sources such as industrial effluents or firefighting training facilities. On the other hand, the presence of PFAS, while generally correlated with the degree of urbanization, is not limited to densely populated areas and rather reflects two distinct mechanisms represented by the identification of persistent long-chain PFAS compounds already phased out and the increasing detection of short-chain PFAS substitutes, due to a progressive shift of the industrial manufacture. However, the impact of these alternatives on the ecosystems and human health still needs to be fully elucidated.

The adverse effects and occurrence of PFAS on aquatic organisms from different trophic levels have been also reported. Indeed, an acute toxicity study conducted with PFOS, PFOA and PFHxS on nine different amphibian species from North America revealed the occurrence of variable toxic effects depending on the type of PFAS, the developmental stage and the species involved. For example, across all the tested species, PFOS exhibited far greater toxicity than PFOA and more severe effects were observed in the salamanders than in frogs or toads, based on the LC_50_ values derived after 96 h of exposure in acute toxicity testing. Also, gray tree frogs were found to be more sensitive to PFAS at later developmental stages while the opposite was true for small-mouthed salamanders, thus suggesting that apart from the type and environmental concentration, species-specific effects can determine the sensitivity of biota to PFAS substances [122]. In another work, male largemouth bass (*Micropterus salmoides*) from Lakes in Minnesota were found to contain variable amounts of PFAS (from 3.2 to 834.4 ng/g fish wet weight) belonging to 13 different classes, among which PFOA was the most represented. Molecular analysis in the liver and testis revealed that the impacted fishes showed dose-dependent alterations in the gene expression, RNA processing, protein turnover, xenobiotics detoxification, lipid and energy metabolism [123]. Similarly, by analyzing the serum concentrations of 23 legacy and novel PFAS in Striped Bass (*Morone saxatilis*) from the Cape Fear River, in North Carolina, Guillette et al. found the presence of 11 different species at variable concentrations. The total PFAS concentration were found to be roughly 40 times higher than those from counterparts grown in an aquaculture field laboratory, with a predominant occurrence of PFOS (mean concentration of 490 ng/mL) followed by PFDA (68 ng/mL), PFNA (4.40 ng/mL) and GenX (1.91 ng/mL). The authors found that the high levels of these and other PFAS were positively associated with increased lysozyme and AST activities, indicating an altered function of the immune system and liver [124].

##### PFAS Occurrence in Marine Water and Sediments

Marine waters cover more than 70% of the Earth surface and represent invaluable sources for many ecosystems due to their key role in the biogeochemical cycles, energy flows and biodiversity. In the same time, oceans are regarded as vital resources for the society and a large fraction of human populations that directly depend on them for their own survival and welfare [125]. Sea waters and oceans constitute major environmental receptors of PFAS substances released and dispersed through multiple mechanisms so that their presence has been largely documented worldwide [126] (see Figure 3). One site of intense investigation has been the Chinese Sea which is part of the Pacific Ocean, and a very important sea lane in the world. For instance, Yan et al. performed a quantitative analysis of C3-C14 PFAS in the riverine and marine sediment samples from estuarine and coastal areas of the East China Sea to reveal an average total PFAS concentration of 9 ng/g dw and a prevalent occurrence of PFOS, followed by PFHpA and PFOA. Of note, the temporal trend of PFOS indicated a clear increase over time as a result of the extensive use over the previous decades and a comparative analysis revealed much higher levels (mean value of 11.4 ng/g dw) than those reported from other countries or regions [127]. A subsequent study collected surface water samples from stations located in the eastern part of the South China Sea, Hong Kong and Macau to show that the extent and composition of PFAS occurrence was strongly influenced by the socio-economic status and most evident in the heavily developed region of the Pearl River Delta. Indeed, while the analysis of the water samples from the South China Sea were characterized by total PFAS concentrations in the range of 0.195–4.925 ng/L, those from Hong Kong and Macau ranged from 2.2–13.6 ng/L. In all the cases, the most represented substances were PFOS, PFOA, PFBA and PFBS but new commercial substitutes, namely 6:2 diPAP and 8:2 diPAP were only detected in Hong Kong and Macau sapling points, at concentrations that were found to be an order of magnitude higher than those reported in a previous study from 2009 [128]. Another area of interest in China has been the Bohai Sea. In a former study from Hong et al., surface water and sediment samples were analyzed for the presence of short-chain and long-chain PFAS over two periods representing the low-water and high-water phases. The overall concentration of PFAS in seawater and sediment ranged from being undetectable to 99.4 ng/L and from 0.33 to 2.78 ng/g dw, respectively. The authors found that PFOA was the predominant analyte in both the samples and also observed high levels of PFBS, PFHxS and PFOS, which however exhibited temporal variations in the sole water samples indicating that a different seasonal activity characterized also the sources of PFAS emission into Bohai Sea [129]. In a later study the same authors better analyzed the multiple inputs of PFAS in the Bohai Sea measuring the PFAS levels in riverwater, coastal wastewater and other effluents which directly flow into this seawater. In the riverwater samples the dominant compounds were PFBS, PFOA and PFOS and the total PFAS levels ranged from 13.1 to 69,238 ng/L revealing the presence of sites with heavy contamination. By contrast, PFOA was the most represented substance in coastal wastewater and effluents, wherein the total PFAS concentration ranged from 16.7 to 7522 ng/L and from 13.1 to 319 ng/L, respectively. The authors concluded that the input from riverine was playing a major role in PFAS contamination of the Bohai Sea, while the discharge of coastal wastewater and effluents contributed to a lesser extent [130]. A subsequent study from the same group further extended these observations by analyzing 21 different PFAS including also novel fluorinated alternatives (Cl-PFESAs) and their seasonal variations in rivers, drain outlets and their receiving Bohai Sea. As a result, severe contamination of the seawater was observed, with total PFAS levels 2–3 orders of magnitude higher than those observed in other areas (between 5.03 and 41,700 ng/L). In general, the authors found that the prevalent analytes were PFOA and PFPeA but significant amounts of 6:2 Cl-PFESA were also detected at levels as high as 4700 ng/L, similar to that of PFOS, indicating that rivers and outlet drains acted as primary sources of PFAS contamination [131]. In the most recent study, a complementary analysis was performed on other environmental media of the Bohai Sea including the coastal water-dissolved phase, surface sediment and suspended particulate matter (SPM). Here, the authors observed total concentrations of PFAS in the water-dissolved phase, surface sediment and SPM between 20.5–684 ng/L, 2.69–25.0 ng/g dw and 4.39–527 ng/g dw, respectively and a clear prevalence of PFOA (mean concentration of 105 ng/L). Of note, PFAS with short carbon chains (e.g., HFPO-DA) were mainly detected in the water-dissolved phase, while long-chain PFAS (e.g PFOA, PFHxA, PFOS, 6:2 and 8:2 Cl–PFESAs) mainly occurred in the surface sediment and SPM phases [132]. Other groups focused on the Pearl River Delta, one of the most highly industrialized and urbanized regions in China, which also represents a major source of PFAS emission into the South China Sea. By collecting samples of surface waters, bottom seawaters, and sediments from 2017 to 2018, Wang et al. found corresponding levels of PFAS between 0.125–1.015 ng/L, 0.038–0.779 ng/L, and 0.0075–0.0842 ng/g dw. PFOA and PFBA prevailed in seawater samples, while PFOS was the most abundant in sediments. For the first time, the authors reported the occurrence of novel PFAS such as HFPO-DA, 6:2 and 8:2 Cl–PFESAs in the South China Sea and their preliminary risk assessment suggesting that PFOS might constitute a low to moderate risk for the marine organisms living in this coastal area [133]. Also, the temporal and spatial distribution of PFAS has been investigated for the first time in Western Mediterranean Sea by Brumosky et al. Here, the analysis of surface water samples revealed the presence of 15 different PFAS substances quite uniformly distributed across the selected area and present at total concentrations in the range of 0.246–0.515 ng/L, consistently with those reported in the Atlantic near the Strait of Gibraltar (2007–2010). Consistently with the local manufacture, uses and regulatory strategies in favor of shorter carbon-chain molecules, PFHxA, PFHpA, PFOA, PFHxS and PFO, were the compounds most frequently detected in all the samples [134]. Interestingly, recent work shed light on the importance of sediments as sink and potential source of PFAS exposure for benthic organisms by analyzing the presence of PFAS substances in the core and surface sediments from the Bearing Sea to the western Arctic Ocean. Here, the authors found a marked prevalence of PFOS, followed by PFNA and PFBS, with a total PFAS concentration in the range of 0.06–1.73 ng/g dw in surface sediments yet subdued to seasonal changes. The major sources were attributed to the oxidation of consumer products containing PFOS, the oceanic transport of fluoropolymer manufacture facilities and the discharge of AFFFs [77]. Lastly, a very recent work for the first time reported the levels of 11 different PFAS in the Eastern coastal waters of the Red Sea, which were above the LOQ and present at concentrations up to 956 ng/L. Among the others, PFHxA, PFHxS, and 6:2 FTS were the most prevalent substances while possible sources of contamination were recognized in discharges from local industries or firefighting training facilities proximal to WWTPs [38]. On the other hand, the presence of PFAS has been documented in a number of different aquatic organisms. Among them, marine plankton from the Northwestern Atlantic Ocean [126] or filter-feeding shellfish from Atlantic and Mediterranean coasts of France, exhibited levels of specific PFCAs (i.e., PFTrDA) as high as 1.36 ng/g ww [39]. Even higher concentrations were attributed to sharks and rays species from Mediterranean Sea, wherein the dominant PFTrDA was detected at levels as high as 27.1 ng/g ww [135]. Importantly, while first acute toxicity studies conducted on marine invertebrates suggest lethal concentrations in the range of 1.1–24 mg/L [136], they appear not to be adequate to predict long-term and sub lethal effects of PFAS requiring the design of novel studies assessing chronic exposure of aquatic organisms. In this regard, one very recent example is represented by a work wherein the long-term effects of the recently introduced compound known as C604 were investigated on the Manila clam (*Ruditapes philippinarum*) exposed to environmentally relevant concentrations (ranging from 100 ng/L and 1000 ng/L) for 7 and 21 days. Here, the authors observed a number of alterations in the transcription profile of genes involved in the regulation of the immune system, neurodevelopment, protein ubiquitination, cell membrane function, lipid and xenobiotic metabolism, indicating that potential ecotoxic effects and risks for marine organisms might be present in certain areas even after PFOA substitution and phase out [137].

##### PFAS Occurrence in Ground Water

Groundwater can be regarded as the sum of all the aquifers located in the saturated zone below the ground surface and constitutes around 30% of world’s freshwater content. From an ecological perspective, groundwater plays a crucial role in maintaining the water level and flow into rivers, lakes and wetlands, representing also a natural resource for the wildlife and plants. In addition, groundwater is extremely important for many socio economic aspects, being the main source of water for industrial processes, agriculture and drinking water. Several sources and pathways of contamination have been described for groundwater reservoirs worldwide largely contributing to indirect PFAS human exposure (see Figure 3). For example, Yao et al. conducted a regional scale investigation on two industrialized cities in the North China to analyze the PFAS levels in the surface rivers and adjacent groundwater. Here, the authors revealed a contribute from point as well as non-point sources leading to variable concentrations of PFAS that were detected at levels as high as 100 ng/L in specific sampling sites, with major contributes of PFOS, PFOA, PFBA and N-EtFOSAA [138]. Chen et al. instead conducted an investigation on different environmental media from eight rural areas in eastern China, revealing the presence of total PFAS levels in the groundwater ranging from 5.3 to 615 ng/L, with highest concentrations occurring in the industrial area responsible of fluorochemical production. The most abundant PFCAs in groundwater were PFBA and PFOA, followed by PFNA, PFHpA, and PFHxA while the prevalent PFAS was PFBS, followed by PFOS and PFHxS [139]. Another study focused on Fuxin fluorochemical industrial park (FIP) in northwestern Liaoning Province of China to determine PFAS occurrence in both surface water and groundwater. Here, Bao et al. observed very high levels of total PFAS, in the range of 216–26,700 ng/L. A marked prevalence of PFBS and PFOA was seen in the groundwater samples, while the relative abundance of these compounds was found to be 24 and 5 times higher than previously reported by the same authors in 2009 [140]. The authors concluded that these PFAS potentially posed serious health risks to local residents, being also detected in home-produced eggs and vegetables [141]. A subsequent study from the same authors revealed a further increase in the levels of PFAS pollution of the groundwater beneath FIP, with concentrations of the dominant PFOA and PFBS up to 2470 and 32,400 ng/L, respectively. The occurrence of significant PFAS levels in greenhouse soils and vegetables led the authors to conclude that the use of contaminated groundwater for agriculture irrigation could have been a major source of PFAS diffusion into human food sources [142]. Another study was conducted in proximity of a landfill located in the northwest of Hangzhou City, in Zhejiang Province, China. Here, the authors found total PFAS concentrations in the groundwater samples ranging from 17.3 ng/L to 163 ng/L. Among the others, the most abundant component was PFBA, followed by PFOA, PFPeA, PFHxA and PFHpA but F-53B and 6:2 FTS were also present at levels up to 5.01 ng/L and 0.52 ng/L, respectively. Despite the authors concluded that the landfill leachate contributed to surface water but not groundwater contamination, representing a low potential risk for human health, they recognized the need of more detailed investigations [143]. More recent work focused on an urban re-development area in Australia characterized by the presence of multiple landfill sites and a high degree of industrial activity. Here, the total PFAS concentrations in the groundwater samples ranged from 26 to 5200 ng/L and the most frequently detected compounds were PFOS, PFOA, PFBS and PFHxS, this latter showing also the highest median concentration (34 ng/L). The authors found a positive correlation between total PFAS, PFOA and other PFCAs (e.g., PFHxA) with typical leachate indicators such as ammonia-N and bicarbonate while this was not true for PFSAs such as PFFO and PFHxS. Based on these data, it was concluded that groundwater contamination was caused not only by the infiltration of landfill leachate but also due to other local and diffused inputs of industrial origin [36].

Groundwater from both industrial and non-industrial areas have been reported to be contaminated by legacy and alternative PFAS compounds, including 6:2 FTS and F-53B at quite high levels [144], as also evidence by a very recent study conducted on the Loess Plateau area, in northwestern China, wherein the presence of legacy and novel PFAS has been investigated. From their analysis, the authors identified a total PFAS concentration ranging from 2.78 to 115 ng/L, and a marked prevalence of PFOA. Also, several emerging PFAS, such as 6:2 FTS, Cl-PFESAs, ADONA and HFPO homologues were frequently detected, while major sources were identified in the industrial activities in the urbanized areas and agriculture activities in the rural areas [145].

Another study assessed the occurrence of PFAS in river and groundwater from several locations along the Ganges River basin in India, revealing contamination with 14 different substances among which the most abundant were PFBA (up to 9 ng/L), PFHxA (up to 4.9 ng/L) and PFHpA (up to 3.5 ng/L). Potential sources of PFAS contamination to groundwater were attributed to river water infiltration, leaching from agricultural soil, municipal, industrial wastewater and landfills [146].

Groundwater pollution can also derive by the use of recycled water from WWTPs for agricultural application, as evidenced by a study from Szabo et al. conducted on the Werribee Irrigation District located around 30 km south-west of Melbourne, Australia. Here, groundwater samples were collected in 2017 and 2018. The data revealed the presence of 20 different PFAS substances, with a total concentration from 0.03 to 74 ng/L and a marked prevalence of PFOS, PFBS, PFOA and PFBA at mean concentrations of 11 ng/L, 4.4 ng/L, 2.2 ng/L and 6.1 ng/L, respectively. Interestingly, the authors found that these levels were comparable to those reported for typical wastewater effluents, higher than those normally observed in the uncontaminated groundwater but much lower than those reported for sites impacted by AFFFs discharge or PFAS manufacture, concluding that the use of recycled wastewater for irrigation of crops was causally responsible of groundwater contamination [147].

Other well documented sources of groundwater contamination are the firefighting training facilities that discharge AFFFs containing PFAS or their precurors, leading to extremely high levels of PFAS in the environmental matrices [61,148]. In this regard, a work from Schultz et al. analyzed the PFAS content in the groundwater from Tyndall (Florida) and Wurtsmith (Michigan) air force bases, wherein fire training activities have been conducted. Here, the total concentrations of 4:2, 6:2, and 8:2 FTSs were reported to be exceptionally high (up to 14.6 × 10^6^ ng/L) in close proximity to Tyndall base, but also very high amounts of total perfluoroalkyl sulfonates (up to 3.5 × 10^6^ ng/L) and perfluoroalkyl carboxylates. Very high levels of total fluorotelomer sulfonates, perfluoroalkyl sulfonates and perfuoroalkyl carboxylates were also observed around Wurtsmith base at concentrations as high as 298 × 10^3^ ng/L, 213 × 10^3^ ng/L and 110 × 103 ng/L, respectively [149]. In another investigation, Houz et al. collected and analyzed groundwater, soil, and aquifer solids samples from a location at Ellsworth Air Force Base in South Dakota, USA, wherein a firefighter training was conducted between 1942 and 1990. Here, median concentrations of PFOS (19,000 ng/L) and PFOA (26,000 ng/L) were observed while similar or higher levels were found for their C6 analogs, PFHxS (71,000 ng/L) and PFHxA (36,000 ng/L). The PFAA precursors, included 6:2 FTS (median concentration of 25,000 ng/L), FHxSA (median concentration of 4000 ng/L), and 8:2 FTS. From their analysis the authors concluded that most of the PFAA precursors originally present in AFFF were transformed into other precursors and PFAAs over a period of several decades [150]. Another study work analyzed different environmental and biological matrices sampled from an area including Oakey, a town which is located in Australia (Queensland) and in close proximity of a fire training facility that made use of PFAA-containing AFFFs since the 1970s. Elevated concentrations were observed in the groundwater, wherein PFOS was the most abundant compound, followed by PFHxS, detected at an average concentration of 4300 ng/L and 2300 ng/L, respectively. Other PFAAs detected in over 50% of the water samples included PFBA, PFPA, PFHxA, PFOA and PFBS all present at mean concentrations between 120 ng/L and 600 ng/L. Further investigation revealed that the use of PFAS containing groundwater led to secondary contamination of human livestock and food, resulting in serum PFOS concentrations that in some cases were almost 30 times higher than those from the general population living in Australia [151]. More recently, Dauchy and coworkers assessed the occurrence of PFAS in groundwater and soil nearby a firefightning training site active for more than 30 years. Interestingly while total PFAS concentrations in the monitoring wells located upgradient from the firefighter training site ranged from <LOQ to 265 ng/L, those located in the perimeter of the firefighter training site or downgradient in the direction of groundwater flow, were characterized by much higher levels, ranging from 300 to 8300 ng/L. Also, 6:2 FTAB was identified in 6 monitoring wells at levels ranging from 45 to 635 ng/L, suggesting that this compound can reach a water aquifer located 20 m below the ground surface and even greater depths, despite the presence of clay layers in the soil [152]. Lastly, while analyzing the presence and distribution of PFAS in the tropical areas constituted by the French Overseas Territories, Munoz et al. observed that groundwater samples exhibited high frequency of short-chain substances, among which the most represented were PFBS, PFHxS, PFOA and PFHxA while PFOS congeners were detected at lower frequencies. Despite this, the authors found that the maximum PFAS levels in groundwater were about one order of magnitude higher than those observed in surface waters reaching values as high as 638 ng/L. Also, high levels of 6:2 FTS and short-chain PFCAs were found in groundwater near several industrial facilities which was attributed to the likely presence of firefighting training facilities at the site, releasing AFFFs [153].

PFAS have been detected in groundwater from different sites worldwide due to multiple local or diffused contamination sources at levels above their limit of quantitation, a reference parameter that has progressively benefited from the introduction of technologies with higher sensitivity [154]. Among the different contamination pathways, the most severe impact derives from the environmental release of AFFFs used in firefight training facilities followed by the discharge of contaminated wastewater in effluents during PFAS manufacture activities. Agricultural use of contaminated irrigation water or leakage from landfill somehow contribute to a lesser extent but is still relevant. Since groundwater can directly impact the consumption of drinking water and food, the presence of PFAS represents a serious concern for human health and more extensive investigation will be needed to better elucidate PFAS impact. Taking also into account the lack of data related to long-term effects of PFAS for human populations subdued to chronic exposure, it is advised that regulatory measures will strongly rely on the precautionary principle to protect groundwater resources for future generations [155].

##### PFAS Occurrence in Drinking Water

Drinking water constitutes one of the most important sources of exposure to PFAS for human populations, since even relatively low levels of PFAS in drinking water can in turn lead to significant burdens in blood serum over a lifetime period [84]. It has been estimated that the prolonged uptake of PFOA through drinking water will result in blood concentrations up to 100 times higher than those observed in the ingested drinking water due to the combined effect of slow clearance and bioaccumulation, compared to other non-human species [156,157,158]. Evidence indicate that a significant proportion of public water supplies, serving up to millions of people, contain PFAS present at levels above the limits set by the national environmental agencies, while potential adverse effects for human health are believed to occur from long-term exposure to drinking water containing PFAS levels even below 70 ppt [159,160,161]. The occurrence of PFAS in drinking water has been documented in different countries worldwide (see Figure 3) and has taken advantage of highly sensitive detection methods such as those based on liquid chromatography-tandem mass-spectrometry (LC-MS/MS) [162] or HPLC coupled to quadrupole time-of-flight high resolution MS (HPLC/QToF-HRMS) [163]. In 2006 Holzer et al. conducted a biomonitoring on people residents in a vast area of North Rhine-Westphaliain, Germany to find elevated PFCs concentrations in blood plasma of children and adults exposed to contaminated drinking water. Among the others, PFOA was the dominant substance in drinking water (500–640 ng/L) and its corresponding plasmatic levels in residents living in Arnsberg were found to be 4.5–8.3 times higher than those for the reference population [164]. Also, Skutlarek et al. assessed PFAS concentration in drinking waters samples derived from public buildings of the Rhine-Ruhr area, in Germany. Here, consistently with the results from surface water samples, the authors identified several PFAS substances in the drinking water with a marked prevalence of PFOA (519 ng/L), followed by PFHpA (23 ng/L) and PFHxA (22 ng/L), thus suggesting that the applied water treatment procedures were not sufficiently effective to remove PFAS from surface waters [165]. In a seminal paper Wilhelm et al. conducted a detailed analysis on drinking water and drinking water resources in the North Rhine-Westphalia (NRW) during the 2008–2009 period, to reveal the occurrence of PFCs at levels ranging from 12 ng/L (i.e., PFHpa) to 1000 ng/L (i.e., PFOS) [166]. In another context, Boiteux et al. analyzed raw and treated water samples collected between 2009 and 2010 in France, showing that PFOS, PFHxS, PFOA, and PFHxA were the most frequently detected PFAS in raw water, with PFHxA exhibiting the highest levels (139 ng/L) among the others [167]. A well-known case of heavy drinking water contamination by PFAS is represented by the Veneto Region, in Italy, where high levels of these substances have been discovered since 2006 [168] and further confirmed in 2013, due to industrial emissions dating back to 1968 [169,170]. Here, in a former investigation, Mastrantonio et al. compared the mortality rate for some causes of death during the period 1980–2013, in residents with similar socio-economic conditions and smoking habits living in municipalities with PFAS contaminated and uncontaminated drinking water. The authors observed that residents from contaminated municipalities had higher relative risks for general mortality, diabetes, cerebrovascular diseases, myocardial infarction, Alzheimer’s and Parkinson’s diseases as well as kidney and breast cancer [171]. These results prompted a later biomonitoring study on different subgroups exposed to contaminated drinking water, revealing that serum concentrations of PFOS and PFOA were above their respective LOQ in all the samples while those related to PFHpA, PFNA, PFDA, PFUndA, and PFHxS were above their LOQ in >50% of the cases. Of note, with the exception of PFUndA, mean concentrations of all the PFAS were higher in the exposed group compared to the not exposed counterparts, with the largest difference observed for PFOA [172]. Few years later, Pitter et al. analyzed serum PFAS levels in adolescents and young adults residing in Veneto (Italy) and exposed to contaminated drinking water. The authors detected significant amounts of only three PFAS substances out of 12 compounds in around 80% of the samples. The main contaminant was PFOA, detected at mean concentrations of 44.4 ng/L, followed by PFOS and PFHxS. The authors concluded that the serum PFOA levels in residents exposed to contaminated drinking water were substantially higher than those observed in other populations with residential exposure [21]. Due to the progressive industrial shift towards alternative substances, new PFAS started to be detected in drinking water. For example, a recent study reported the presence of one PFOA substitute known as HFPO-DA and one catalyst used in the production of polymers, known as F_3_-MSA, in Dutch and Belgian waters. Here, HFPO-DA was detected in 46% of the drinking water samples at relatively low mean levels (2.9 ng/L) despite in some sites (i.e., Lekkerkerk-Tiendweg) values as high as 28 ng/L were seen. By contrast, F_3_-MSA was detected with higher frequencies (68.2% of the samples) and at higher concentrations, (average and maximum concentration of 24 ng/L and 165 ng/L, respectively), suggesting a lower removal efficiency by drinking water treatment [173]. In Sweden, a quite recent study focused on adults living in Ronneby, a municipality where one water work had been heavily contaminated from PFAS contained in AFFFs. Already in 2013 the analysis of drinking water reservoirs revealed the presence of PFOS, PFHxS, PFHxA, PFBS and PFOA at concentrations up to 8000 ng/L, 1700 ng/L, 320 ng/L, 130 ng/L and 100 ng/L, respectively. From an extensive biomonitoring, the authors observed that the blood levels of PFAS in the exposed groups were up to 100 times higher than those from the controls, with mean concentrations of PFOS, PFHxS and PFOA equal to 157 ng/mL, 136 ng/mL and 8.6 ng/mL [174]. Later reports assessing the blood PFAS levels in people living in the same municipality revealed that the means for serum PFHxS, PFOS and PFOA for all Ronneby residents were 135, 35 and 4.5 times higher than those seen in the control group [41].

Several studies have also reported PFAS contamination of drinking water in a number of countries outside EU. For instance, Hu et al. presented a thorough analysis conducted during 2013–2015 by the US EPA UCMR3 program on PFAS occurrence in national drinking water. Here, the content of PFOA and PFOS in drinking water supplies serving almost 6 million of residents, exceeded the advisory threshold of 70 ng/L, raising serious concern for potential adverse effects, further exacerbated by the absence of information on private wells that represented up to one third of U.S. population drinking water supplies [159]. Another report from Sun et al. analyzed the contamination status of the Cape Fear River watershed in North Carolina, which is a fundamental source of drinking water for three distinct communities living in that area. Here, in communities A and B, only legacy PFAS were detected at a mean total concentration of 355 ng/L and 62 ng/L, respectively, with marked prevalence of PFHxA, PFPeA, PFHpA PFOS, PFOA and PFBA. By contrast, in the community C living downstream a PFAS manufacture facility, the authors observed relatively low amounts of legacy PFAS but very high concentrations of GenX reaching levels close to 4500 ng/L [175]. Also, Tan and coworkers assessed the PFAS occurrence in the tributary system of the Yangtze River in correspondence of the Jiujiang Province, in China. Here, the analysis of tap water samples revealed the presence of total PFAS levels in the range of 2.4–290 ng/L, with the highest concentrations observed at Jiujiang City. Among the others, PFOA was the predominant compound followed by PFBA or PFNA, while PFBS was absent. By comparing these values with those reported from tap water in eastern China (range of 1.4–175 ng/L) [176] and the US-EPA guidelines for PFOA and PFOS levels in drinking water, the authors concluded that the health risk for the residents the Jiujiang city were relevant [113]. Another quite recent work from Guardian et al. assessed the occurrence of PFAS in drinking and surface water from Thailand and the Philippines. Here, 12 different PFAS were detected in the drinking water samples with total PFAS concentrations ranging from 7.16 to 59.49 ng/L and from 9.08 to 11.63 ng/L, respectively in samples from Thailand and Philippines. Among the legacy substances, PFOA, PFOS, PFNA and PFHpA, were detected in all water samples from both countries, the latter compound being the most abundant. Of note, for the first time the authors reported the occurrence of novel PFAS alternatives such as N-MeFOSAA, which was the second most abundant compound in both the countries [177]. Also, by assessing the drinking water source known as Baoshan Reservoir, located in Hsinchu City, Taiwan, Jiang et al. identified 6 different PFAS substances in all the tested samples. The most frequently detected were PFOA (32.4%), PFOS (28.8%), PFNA (15.7%), PFHxS (14.4%), PFHpA (4.92%) and PFBS (3.74%), with maximum concentrations of 68.9 ng/L, 61.2 ng/L, 33.4 ng/L, 30.5 ng/L, 10.5 ng/L and 7.94 ng/L, respectively. Despite the authors formally calculated that these values resulted in a low risk for human health using the USEPA reference dose, they did not exclude potential adverse effects [178].

Taken together these data indicate that PFAS contamination of drinking water sources and tap water is widespread across different countries. Despite the presence of variable sources, some PFAS compounds such as PFOA and PFOS are still present at significant concentrations despite they have been subdued to strict regulatory measures or phased out. Apart from this, shorter-chain substitutes such as HFPO-DA (GenX), PFHxA, PFHxS or PFBA start to be detected in drinking water sources and in serum levels of human populations with an alarming frequency. The lack of thorough epidemiological and observational studies for all these compounds is a major shortcoming in the assessment of their impact on human health. Even when appropriate epidemiological and biomonitoring studies are available, as in the case for legacy PFAS, the paradigmatic methods used to calculate the risks for human subpopulations are subject to significant uncertainty, potentially leading to an underestimate of the real impacts of PFAS substances. This is especially true for example when the PFAS levels fall below the formal Health Index or do not exceed the threshold for the specific advisory limits, while the lack of uniform guideline values adopted in different states/regions is a further complication [58]. Also, it is important to note that most of the regulatory guidelines for drinking water are related to PFOA and PFOS while other legacy PFAS and emerging alternatives are increasingly detected in water samples. Another important aspect, which is frequently overlooked is that PFAS are almost invariantly present as mixtures (sometimes even with poorly defined composition) and coexist with other pollutants, leading to chronic exposure (up to several years) and progressive bioaccumulation. Based on this, it is recognized that current experimental strategies are not entirely adequate to unveil the real toxic potential of these substances, especially regarding the potential lifetime exposure, thus requiring more refined testing strategies. For this reason, in absence of toxicological data on possible synergistic effects, it cannot be excluded that serious adverse effects might occur in human populations consuming large amounts of PFAS-contaminated drinking water in the long-term, suggesting the need for a new paradigm in the evaluation of PFAS toxicity.

#### 2.4.3. PFAS Occurrence in Soil and Plants

With few exceptions, PFAS substances are released into the environment through subsurface sources and almost invariantly impact on soils where they undergo adsorption, volatilization, biotransformation, speciation and uptake processes, before they move into other compartments such as surface or groundwater systems (see Figure 3). The prevalent sources of PFAS contamination in the vadose (unsaturated) zone of terrestrial compartments are represented by the atmospheric deposition, the release of AFFFs, the use of contaminated water for irrigation and the application of biosolids or municipal sludge in the agriculture [179] (see Figure 3). Intuitively, the highest degree of soil contamination by PFAS has been observed in the most industrialized countries such as USA, China, or Japan, with reported total concentrations of PFAS as high as 13,000 pg/g dw, 14,000 pg/g dw and 36,000 pg/g dw, respectively [180]. PFAS emissions can become extremely relevant also for human food safety when point sources such as fluoropolymer manufacture facilities are close to agricultural lands, leading to high concentrations of total PFAS in the soil (up to 200 ng/g) and even higher levels in edible crops (up to 8085 ng/g) due to bioaccumulation mechanisms [181].

##### PFAS Occurrence in Soil through Atmospheric Dispersion

Airborne dispersion can be a significant transport pathway through which neutral volatile PFAS substances (such as FTOHs, FOSAs and FOSEs; 6:2 FTOH, 8:2 FTOH, 10:2 FTOH, MeFOSA, MeFOSE, EtFOSA and EtFOSE) or even ionic PFAS with high affinity for the particulate matter (such as PFOA, PFOS, PFNA, PFHxS and PFBA), can potentially migrate from the initial emission source to distant sites and eventually be deposited on top soil [182,183] (see Figure 3). A seminal study from Rankin et al. assessed the PFAS occurrence in surface soils from 62 different locations representative of all continents wherein direct human activity was absent, thus representing an important reference for background values in soil compartment. Here, the authors found that PFCAs were present in all the soil samples at total concentrations ranging from 29 to 14,300 pg/g dw while PFSAs (PFHxS, PFOS and PFDS) ranged from <LOQ to 3270 pg/g dw and were observed in the vast majority of the samples. Additional analysis revealed that PFOA and PFOS were the most frequently detected compounds, with concentrations up to 2670 and 3100 pg/g dw, respectively. The authors concluded that several PFAS were widely distributed across different countries, reaching detectable levels even in absence of evident human activities, most likely due to mechanisms of long-range transport and back deposition [184]. Similar mechanisms cause also the transport of PFAS substances from a point source, as evidenced by a study from Chen et al. wherein the composition and distribution of PFAS compounds was analyzed in an area surrounding two fluoropolymer manufacture parks located in Fuxin, China. As expected, the total PFAS concentrations close to the plants were 1–2 orders of magnitude higher than those detected far from the sites, ranging from 2.4 to 240 ng/g dw. PFBA and PFHpA were the most abundant compounds in all the soil samples while 8:2 FTUCA and FTOHs were found only close to one plant. Of note, the authors hypothesized that PFHpA in soil might originate from a biotransformation process of FTOHs rather than direct emission. The authors also observed that the levels of PFBA (up to 14,000 ng/g dw), PFOA (up to 1500 ng/g dw) and PFOS (up to 930 ng/g dw) in plant leaves close to the point sources were much higher than those from the respective soil samples, revealing extensive uptake and bioaccumulation of these substances [185]. In another recent study, Galloway et al. analyzed the presence of PFOA and its replacement substitute in surface water and soil samples from an area located in Ohio and West Virginia close to a fluoropolymer manufacture facility. Here, the authors found detectable levels of PFOA in soil samples from 2016 and 2018, ranging from 5 to 27 ng/g, but also concentrations of HFPO-DA above the LOQ (though around 1 order of magnitude lower than PFOA concentrations) at five distinct sites, suggesting long-term contamination of the soil matrix [186].

##### PFAS Occurrence in Soil through AFFFs Discharge

A number of studies have assessed the occurrence of PFAS in surface soil upon intentional or accidental release of AFFF-containing products into the environment [187,188] (see Figure 3). From these and other investigations a clear prevalence of perfluorinated sulfonates, generally between C4-C8 carbon chain length, have been observed in soil samples from AFFF impacted sites, with PFOS and PFOA reaching concentrations as high as 6500 µg/kg, depending on the entity of soil contamination. Of note, two important aspects of this type of contamination were revealed. Indeed, in one case the data indicated that PFAA precursors contained into the AFFF formulations had been transformed into other precursors and PFAAs over the decades of persistence in the subsurface through speciation, oxidation or biotransformation mechanisms [150]. Besides, other data also indicated that the extent of soil contamination can influence the entity of PFAS infiltration below the ground surface, since low impacted sites had measurable PFOS levels between 0–2 mt of depth while in highly impacted sites, the soil contamination extended up to 4 mt below the surface, implying different mechanisms of attenuation and transport [189].

##### PFAS Occurrence in Soil through Irrigation Water

Some authors have also focused on the use of contaminated irrigation water, revealing that this source can potentially affect the agricultural soil and thus plants or vegetables that are part of the human diet (See Figure 3). For example, a recent study assessed the contamination status of the Nakdong River in South Korea over 2013–2017, revealing the presence of PFOA and PFOS at annual mean concentrations ranging from 0.026 to 0.112 µg/L (irrigation water), and from 0.818 to 1.364 µg/L (soil), respectively, exceeding the Californian OEHHA’s advisory guidelines for inland surface water. The authors also documented that relevant concentrations of PFOA and PFOS could be found in six different edible crops, including leafy vegetables, fruits and rice, whose uptake significantly contributed to human PFAS exposure [190]. Consistently, other data suggest that the use of contaminated groundwater for agriculture in sites located in close proximity to fluorochemical facilities, as the FIP in china, can adversely impact home-produced vegetables and eggs, leading to high concentrations of PFBA, PFBS and PFOA [141].

##### PFAS Occurrence in Soil through the Application of Biosolids

Apart from the use of irrigation water, another important source of soil contamination by PFAS that can directly impact on human food sources derives from the application of biosolids (i.e., sludge produced from WWTPs) or fertilizers for agricultural purposes (see Figure 3). In this respect, quite recent estimates indicate that around 4.5 million tons of biosolids are applied every year to the European soil while these amounts reach an impressive value of almost 7 million tons in the USA [191,192]. Unfortunately, wastewater treatments are not able to efficiently remove PFCA and PFSA compounds and can potentially promote the conversion of polyfluorinated substances into different PFAAs, moreover inducing PFAS accumulation in the solid phase of residual sludge [193,194]. The use of biosolids to amend agriculture lands is a broadly diffused practice and its effects on soil contamination have been documented worldwide. For instance, Washington et al. focused on an area near Decatur, Alabama (USA) to compare the levels of PFAS in soil samples in presence or absence of sludge application. Here, much higher PFAS concentrations were seen in the surface soil samples receiving contaminated sludge, with total concentrations up to around 5 μg/g dw, revealing also the occurrence of FTOHs degradation into additional PFAS congeners [195]. In another study the group of Higgins assessed different agricultural soil samples that had been augmented with municipal biosolids, demonstrating the presence of several PFAS substances among which PFOS was the dominant PFC with concentrations ranging from 2 to 483 ng/g and a proportional increase with the extent of biosolids loading rate [196]. More recent investigations indicate that in addition to legacy PFAS, also new substitutes start to be detected in the sludge, soil and vegetables grown in amended agricultural land. In this respect, Semerad et al. assessed a number of samples from Czech Republic WWTPs, to find high PFAS content in the sewage sludge, with total concentrations up to 9329.9 ng/g, a clear prevalence of PFOS, (detected in ~60% of the samples) and the occurrence of short-chain PFAS substitutes, including GenX, in ~20% of the samples. Predicted theoretical concentrations in the sludge amended soil and vegetables grown in fertilized agriculture land, led to comparable levels in the former and significantly higher values in the latter, particularly in case of celery shoots and lettuce leaves [16].

##### PFAS Impact on Terrestrial Organisms and Human Exposure

Once deposited on soil, PFAS substances can subsequently leach into groundwater, be transferred to surface watersheds or enter into terrestrial food webs including plants, terrestrial organisms, livestock and agricultural products representing key food sources for human diet, thus largely contributing to the human exposure via the environment [74,146]. Accumulating evidence suggest that PFAS soil content is directly correlated with their bioaccumulation rate in plants, which is dictated by the specific chemistry of the PFAS substance, the plant species, the target organs (e.g., root, leaves) and the occurrence of abiotic factors (e.g., temperature, salinity, pH changes, content of organic carbon) influencing PFAS bioavailability [72]. In this respect, PFOA, PFOS and other PFCA or PFSA homologues have been the most widely investigated compounds worldwide [197,198,199], though other substances such as FTOHs, FOSAs, FOSEs and FTAs have been also detected in heavily contaminated sites [200,201]. Effects on terrestrial invertebrates have been reported in a recent study wherein the accumulation and the effects of PFAS were investigated in the earthworm *Eisenia fetidi* exposed to spiked-soil containing increasing amounts of PFNA, PFHxS, PFHpA and PFBS, reflecting the levels typically found in background, biosolid amended and industrially contaminated soils. Here, the authors found that some PFAS were efficiently uptaken by earthworms even under conditions of relatively low exposure. More in detail soil concentrations of PFNA as low as 0.1 µg/Kg resulted in earthworm levels of 13 µg/Kg while the exposure to 1 µg/Kg of soil-containing PFNA and PFHxS led to earthworm concentrations of 32 and 34 µg/Kg, respectively. Starting from 10 µg/Kg of PFAs soil contamination also PFBS and PFHpA were markedly uptaken. Of note, while the authors did not observe obvious effects on earthworm survival or weight loss, at PFAS soil concentrations lower than 100,000 µg/Kg, they also speculated that adverse effects cannot be excluded upon longer exposure time [202]. In another study, Zhang et al. investigated the distribution of PFAS substances in plant-soil-water systems, by studying the bioaccumulation of eight distinct PFAAs in the aquatic macrophyte *Juncus effusus* grown for 21 d in a greenhouse and exposed to different concentrations of these compounds. Here, the authors observed a trend of enhanced root and shoot uptake with increasing PFAA carbon chain length and exposure time. Also, PFSAs were more efficiently uptaken than PFCAs and longer chain PFAAs reached higher concentrations in the root and shoots than the shorter chain PFAAs, in general leading to the conclusion that ~82.8% of all PFAAs were translocated to the soil and plant in 21 days. Importantly, by sequencing the 16 s ribosomal DNA from microbial communities associated with this system, the authors found that the bacterial communities in the controls had higher diversity and richness than those treated with PFAAs, whose effect caused a dose-dependent alteration in the species composition and structure of the bacterial communities [203]. In summary these data clearly indicate that PFAS have been detected in soils from a great variety of different regions worldwide, even in remote regions located at great distance from primary sources of release. Potential long term effects of PFAS soil contamination are extremely relevant for human health due to multiple routes of exposure that drive PFAS redistribution into the atmosphere, drinking water reservoirs and other food sources such as vegetables. Also, serious ecotoxicological effects on terrestrial organisms and plants can be envisaged, although additional investigations will be necessary to understand the interrelation between PFFAS environmental fate and toxicity, with a particular emphasis on the relative contribute of site-specific factors and other environmental conditions.

## 3. PFAS Human Exposure and the Potential Effects for Human Health

It is well recognized that multiple exposure pathways can link PFAS emission from the primary or secondary sources to human receptors represented by professional workers as well as general population. Some of the most relevant exposure routes include the inhalation of air and dust particulate, the ingestion of contaminated food and drinking water and the dermal adsorption [204]. Despite the presence of some gaps in our understanding, it is generally accepted that the dietary intake and the consumption of drinking water represent major pathways for the general population [205,206] while the relative contribute of inhalation and dermal contact is far more relevant in case of occupational exposure [207,208,209]. It is also believed that PFOS, PFOA, PFNA and PFHxS are the PFAS species that currently contribute most to human exposure, a reason for which provisional measures limiting their daily intake have been proposed in 2020 by the European Food Safety Authority (EFSA) [75]. Irrespectively to the specific exposure pathway through which they can get in contact with human targets, PFAS substances represent a serious concern for human health potentially inducing alterations in the development, lipid metabolism and endocrine system, cancerogenicity, immunotoxicity, hepatotoxicity and reprotoxicity. In terms of PFAS risk assessment, the EFSA CONTAM Panel was the first international scientific body to include the data from epidemiological studies to derive health-based guidance values for PFOA and PFOS in 2018. The critical effects of PFOS were identified as a rise in blood total cholesterol in adults and a reduction in antibody response to immunization in children. On the other hand, the key consequence of PFOA was a rise in blood total cholesterol. Reduced birth weight (for both chemicals) and an increased incidence of elevated blood levels of the liver enzyme alanine aminotransferase (ALT) (for PFOA) were also taken into account. Tolerable weekly intake (TWI) of 13 ng/kg body weight (b.w.) per week was defined for PFOS and 6 ng/kg b.w. per week for PFOA after benchmark modelling of serum levels of PFOS and PFOA and estimated the associated daily intakes. It was also observed that the exposure to a significant fraction of the population to both chemicals exceeded the proposed TWIs EFSA (2018) Risk to human health related to the presence of PFOS and PFOA in food [210]. The ATSDR (Agency for Toxic Substances and Disease Registry 2018) also included a decreased antibody response to vaccines (PFOA, PFOS, PFHxS, and PFDA) and increased risk of asthma diagnosis (PFOA) among the list of adverse health effects in PFAS-exposed humans (ATSDR Toxicological Profile for Perfluoroalkyls). Furthermore, the International Agency for Research on Cancer (IARC) classed PFOA as “possibly carcinogenic to humans” (Group 2B), based on limited evidence in humans that it might cause testicular and kidney cancer, as well as limited data in animal studies [211].

Potential of PFAS to cause a wide range of negative health impacts depends of various factors, such as the conditions of exposure (dose/concentration, duration, route of exposure, etc.) and characteristics associated with the exposed target (e.g., age, sex, ethnicity, health status, and genetic predisposition) [212]. The list of biological functions impacted by PFAS in females and males is rapidly expanding. Endocrine disruptive effects have been reported to affect fertility, body weight control, thyroid and mammary gland function. Developmental effects have been observed in children such as alterations in the behaviour or accelerated puberty but also in the new-borns such as decreased birth weight. Increased risk of kidney, prostate and testicular cancer has been associated with long-term exposure to PFAS in the general population alongside with disturbances in the cholesterol metabolism or reduced efficiency of the immune system against infections.

To illustrate this, in the following paragraphs we will describe more in detail some of the adverse effects caused by PFAS compounds, covering some of the recent in vitro, in vivo and epidemiological studies that have been published in the last ten years.

### 3.1. In Vitro Studies on PFAS Effects

Selected in vitro studies (published since 2010) exploring toxic effects of PFAS are summarised in Table 4. Majority of the presented in vitro studies investigated the impact of PFOA and/or PFOS, while the main observed effects were on thyroid [213,214,215,216] and hepatic cells [217,218,219,220,221]. In vitro exposure of thyroid cells to different PFAS was shown to have varied thyroid-disrupting effects. Conti et al. (2020) exposed thyroid follicular cells to 1–100 mM PFOS or PFOA, concluding that both substances acutely and reversibly inhibited iodide accumulation by FRTL-5 thyrocytes. Additionally, PFOS prevented sodium iodide symporter-mediated iodide uptake and reduced intracellular iodide concentration in iodide-containing cells. However, this substance did not affect iodide efflux from thyroid cells [213]. Furthermore, Song et al. (2012) documented decreased TPO activity in FTC-238/hrTPO/RSK008 cells after the exposure to different PFOS and PFOA concentrations [214]. At concentration of 10^5^ nM PFOA/PFOS, a significant inhibition of cell proliferation was found in rat thyroid cell line-5 (FRTL-5), which mostly occurred due to the increased cell death. The results of this study also suggested that PFOA and PFOS enter thyroid cells by a gradient-based passive diffusion mechanism [215]. Croce et al. (2019) investigated the impact of different long-chain and short-chain PFAS, including PFOS, perfluorobutanesulfonic acid (PFBS), perfluorobutanoic acid (PFBA), perfluorophosphonic acid (PFPA) and perfluoropentanoic acid (PFPeA), on the same cell line (FRTL-5), at various concentrations (up to 100 μM). However, aside from PFOS (100 μM), neither long, nor short-chain PFCs impacted cell survival or interfered with cAMP synthesis. As a result, the authors came to the conclusion that short-chain PFCs had no acute cytotoxic effect on thyroid cells in vitro [216].

Some of the in vitro studies explored liver toxicity of the PFAS by investigating the occurrence of the oxidative stress on different hepatic cell lines [218,219], as well as apoptosis/autophagy [219] and cholesterol/bile acids level [221]. The impact of PFOS, PFOA, PFHxS, PFNA, PFDA, PFUnA, and PFDoA on the same cell line (HepG2) has been tested. Here, PFHxS, PFOA, PFOS, and PFNA caused a dose-dependent increase in DNA. With the exception of PFDoA, all other PFAS elevated ROS formation. Cells exposed to PFOA had significantly lower total antioxidant capacity (TAC) compared to the control, but PFOS and PFDoA had a non-significant trend in TAC decrease and an increasing tendency for PFHxS, PFNA, and PFUnA [218]. Other studies also explored the link between PFOA/PFOS and oxidative stress/apoptosis in different liver cells. Zeng et al. (2021) tested the influence of PFOS on human embryo liver L-02 cells. Reduced cell activity was observed, as well as increased ROS levels in a concentration-dependent manner. Impaired mitochondrial membrane potential (MMP), as well as elevated autophagy and apoptosis were observed, together with the increased expression of Bax, cleaved-caspase-3, and LC3-II. The authors concluded that ROS-dependent autophagy might be the cause of PFOS-induced apoptosis in L-02 cells [219]. Other mechanisms underlying PFAS-induced liver injury were also investigated. Human HepaRG liver cells were treated to PFOA, PFOS, and (PFNA at various doses. All PFAS increased cellular triglyceride levels while having no effect on cholesterol levels. In HepaRG cells, PFOA, PFOS, and PFNA enhanced triglyceride levels while inhibiting cholesterogenic gene expression. The authors noted that PFAS-induced endoplasmic reticulum stress might be an essential mechanism underpinning some of the harmful effects caused by these compounds [220]. Similarly, Behr et al. (2021) investigated the effects of PFOS/PFAS exposure and found that cholesterol levels in the same cell line (HepaRG) were not affected by neither PFOA nor PFOS. However, in this study, both substances strongly decreased synthesis of a number of bile acids. Moreover, the expression of numerous genes whose products are involved in synthesis, metabolism and transport of cholesterol and bile acids was strongly affected by PFOA and PFOS at concentrations above 10 µM. Indeed, PFOS and PFOA led to a strong decrease of CYP7A1, the key enzyme catalysing the rate-limiting step in the synthesis of bile acids from cholesterol, both at the protein and the mRNA level. Both substances led to a dilatation of bile canaliculi [221].

Oxidative stress and apoptosis/autophagy were also investigated in the light of PFAS-induced neurotoxicity. Li et al. (2017) exposed rat primary hippocampal neurons and astrocytes to PFOS, which led to redox imbalance, increased apoptosis and abnormal autophagy. In astrocytes, PFOS altered extracellular glutamate and glutamine concentrations, decreased glutamine synthase activity and impaired gene expression of glutamine synthase as well as glutamate and glutamine transporters [224]. Other PFAS-linked neurotoxic mechanisms included interferences at the gene expression level and calcium homeostasis. Wan Ibrahim et al. (2013) investigated the effect of PFOS on primary rat embryonic neural stem cells (NSCs) and noted an increase in neuronal differentiation. Upregulation of PPARγ was also observed, with no changes in PPARα or PPARδ genes. Additionally, PFOS upregulated mitochondrial uncoupling protein 2 (UCP2) and induced Ca^2+^ activity [225]. Both PFOS and PFOA were found to accumulate in cultured neurons and elevate calcium concentrations via release of intracellular calcium stores. 1,4,5-trisphosphate receptors (IP3Rs) and ryanodine receptors (RyRs) were found to take part in PFOS or PFOA induced calcium release, which caused a perturbation of calcium homeostasis [222]. PFOS and PFOA also inhibited the GABA-evoked current in primary rat cortical cultures and acted as non-competitive human GABA-A receptor antagonists. Network activity of rat primary cortical cultures increased following exposure to PFOS [223]. Pierozan and Karlsson (2021) studied the effects of PFOS and PFOA on primary neurons and NSC from rats. There were no impacts on cell viability or proliferation in primary neurons. At the lowest studied concentration (1–100 μM), PFOS exposure boosted NSC proliferation, while PFOS and PFOA altered the morphology of NSC-derived neurons. The neurite network was similarly impacted by 1 and 10 μM PFOA exposure, resulting in an increase in the number of processes and branches per cell. The authors concluded that NSC, which mimics the embryonic brain, are more vulnerable to PFOS and PFOA exposure than neurons [226].

PFAS effects on reproductive function were also investigated in in vitro studies. Eggert et al. (2019) assessed the effects of 0–100 μg/mL PFOA on foetal rat testes and adult rat seminiferous tubule segments. The findings revealed lower levels of cAMP, progesterone, testosterone, and StAR expression. The number of diploid, proliferative, meiotic, and G2/M-phase cells in adult rat testis was drastically reduced by PFOA. By contrast, PFOA had no effect on foetal, proliferating, or adult rat Sertoli cells. Foetal Leydig cells, on the other hand, showed an increased susceptibility to apoptosis [227]. The exposure of human cell lines such as MCF-7, H295R, LNCaP and MDA-kb2PFOA to PFOS and six substitutes including PFHxS, PFBS, PFHxA, PFBA, PMOH and ADONA has revealed that PFOA, PFOS and PMOH enhanced 17β-estradiol-stimulated estrogen receptor β activity, and PFOS, PMOH, PFHxA and PFBA enhanced dihydrotestosterone-stimulated androgen receptor activity. PFOA and PFOS slightly enhanced estrone secretion, and progesterone secretion was marginally increased by PFOA. All these effects were only observed at concentrations above 10 μM [228].

### 3.2. In Vivo Studies on PFAS Effects

PFAS exposure has mostly been linked to adverse outcomes in mice and rats, while the majority of studies explored the toxicity of PFOS, PFOA and PFHxS. Selected in vivo studies (published since 2010) investigating toxic effects of PFAS are presented in Table 5.

A link between PFOS exposure and hepatic lipid metabolism was found in vivo. After the exposure of C57BL/6 mice to 10 mg PFOS/kg b.w./day by oral gavage for 14 days, 241 proteins involved in lipid and xenobiotic metabolism in liver were found to be dysregulated. 16 overexpressed glycoproteins were associated with neutrophil degranulation, cellular responses to stress, and protein processing in the endoplasmic reticulum (ER) [230]. Li et al. (2021) also documented the ability of PFOS to accumulate in the liver of female BALB/c mice after the exposure to 100 μg/kg b.w./day and 1000 μg/kg b.w./day by oral gavage for 2 months. PFOS accumulated in the lungs, kidneys, spleen, heart and brain as well, causing damage in the liver and in the marginal area of the heart. PFOS mainly affected glycerophospholipid metabolism and sphingolipid metabolism in liver. These authors suggested that the upregulated ceramide and lysophosphatidylcholine (LPC) might lead to liver cell apoptosis, while decrease in liver triglyceride (TG) content might result in insufficient energy and cause liver morphological damage [231]. After the dietary exposure of rats to 20 or 100 ppm PFOS for 7 days, Elcombe et al. (2012) have also noted alterations in various liver parameters (e.g., increased liver weight; decreased plasma cholesterol, alanine aminotransferase, and triglycerides; increased hepatocellular cytosolic CYP450 concentration; increased liver activity of acyl CoA oxidase, CYP4A, CYP2B, and CYP3A; increased liver proliferative index and decreased liver apoptotic index). However, in the aforementioned study, thyroid parameters (histology, apoptosis, and proliferation) were not unaffected [236].

Owumi et al. (2021) investigated hepatic and kidney function of PFOA. After the exposure of rats to PFOA (5 mg/kg b.w./day) for 28 days, the authors noted an increase in hepatic (GGT, ALT, AST and ALP) and renal function (urea and creatinine) as well as biomarkers of toxicity, paralleled by a decrease in the activity of the enzymatic antioxidants (CAT, GPx, SOD) in liver and kidney tissue. In this study, a significant increase in lipid peroxidation and pro-inflammatory cytokine IL-1β in rats’ liver and kidney occurred, while a decrease of the anti-inflammatory cytokine, IL-10 was also observed [232]. Similarly, renal damage was found in mice after the exposure to PFOA. Rashid et al. (2020) noted an increase in Dnmt1 with decreased Rasal1 expression at higher levels of PFOA exposure. Rasal1 hypermethylation (an early indicator of fibroblast activation in kidney) was also observed, followed by the increase in Hdac1, 3 and 4, class I & II HDACs which are known to be critically altered in some renal diseases. Furthermore, mRNA levels of TGF-β and α-SMA were significantly increased [233].

Health effects of PFHxS have also been investigated in animal studies. After the oral exposure of F0 and F1 CD-1 mice to 3 mg PFHxS/kg b.w/day, equivocal decrease in live litter size at 1 and 3 mg/kg b.w./day was noted, as well as adaptive hepatocellular hypertrophy, concomitant decreased serum cholesterol and increased alkaline phosphatase [234]. After treating rat with 0.05, 5 or 25 mg/kg b.w./day PFHxS from gestation day 7 onward to postnatal day 22, Ramhøj et al. (2020) observed a dose-dependent decrease in thyroid hormone levels in both dams and offspring, while TSH levels, weight, histology, or expression of marker genes of the thyroid gland were unaffected [235]. Other neurotoxic effects of PFHxS have been investigated as well. Sim and Lee (2022) have found that PFHxS causes long-term developmental neurotoxicity as well as downregulation of GAP-43 and CaMKII via the NMDA receptor-mediated PKCs (and)-ERK/AMPK pathways in mice after the neonatal exposure, together with significant memory impairment in adult mice [236].

### 3.3. Human Studies on PFAS Effects

Selected human studies (published since 2010) investigating toxic effects of PFAS are presented in Table 6. Majority of the human studies explored the linkage between PFAS concentration and lipid status, mainly cholesterol level [237,238], while a study was also conducted to assess the connection between PFAS and cholesterol at the gene expression level [239]. Eriksen et al. (2013) discovered substantial positive relationships between PFOS, PFAS, and total cholesterol in 753 individuals, while sex and prevalence of diabetes were suggested to influence the connection between these two substances and cholesterol [237]. Fletcher et al. (2013) observed an inverse relationship between serum PFOA levels and the expression level of genes involved in cholesterol transport in whole blood (NR1H2, NPC1 and ABCG1). A positive correlation was found between PFOS and a transcript involved in cholesterol mobilization (NCEH1), while a negative relationship was seen between PFOS and a transcript involved in cholesterol transport (NCEH2). Sex-specific effects were also noticed in this study [239]. On the other hand, in a study involving 815 participants ≤18 years of age, Geiger et al. (2014) found that serum PFOA and PFOS were related with high total cholesterol and LDL-C levels, regardless of age, gender, race-ethnicity, body mass index, yearly family income, physical activity, or serum cotinine levels. PFOA and PFOS were not shown to be substantially linked with aberrant HDL-C and triglyceride levels [238].

Other studies explored the link between PFAS concentration and different hormones, such as thyroid [242,247] and sex hormones [245,247,248], as well as development [243,245]. By assessing the connection between the levels of 14 PFAS in healthy men from the general population and different sex hormones and semen sample quality, Joensen et al. (2013) found that only PFOS levels were negatively associated with testosterone, calculated free testosterone (FT), free androgen index (FAI) and ratios of T/LH, FAI/LH and FT/LH. Other PFAS were found at lower levels than PFOS and did not exhibit the same associations [241]. Also, after measuring PFAS levels in 1682 males and females 12 to 80 years of age, Lewis et al. (2015) found no significant relationships between any of the PFAS and testosterone. PFAS were suggested to be associated with increases in FT3, TT3, and FT4 among adult females. The authors concluded that, during the adolescence, PFAS may be related to increases in TSH among males and decreases in TSH among females [242], suggesting sex-specific effects. In contrast, Li et al. (2021) discovered no associations between PFAS and thyroid hormones in adults and seniors in 3297 participants from Ronneby, a municipality with highly contaminated drinking water by PFAS (exposed group), with the exception of a positive association between PFAS and fT4 in males over 50. Thyroid hormone levels were observed to be higher in Ronneby preteen children compared to the control group. Weak evidence of a link between increasing PFAS levels and lower fT3 in preteen boys and lower TSH in adolescent men was found [247].

Lopez-Espinosa et al. (2011) aimed to investigate whether PFOS and PFOA were linked to the markers of sexual development. Their study included 3076 boys and 2931 girls aged 8–18 years. For boys, there was a link between increased PFOS and a lower chance of reaching puberty. Higher PFOA or PFOS concentrations in girls were related to a lower risk of post menarche [243]. The same group of researchers examined the link between PFAS levels and estradiol, total testosterone, and IGF-1 in 2292 children. In boys, PFOA concentrations were substantially related to testosterone levels; PFOS concentrations were related to estradiol, testosterone, and IGF-1, while PFNA concentrations were linked to IGF-1. Significant linkage was discovered in girls between PFOS and testosterone and IGF-1, as well as PFNA and IGF-1 [244]. Furthermore, Wang et al. (2019) concluded that PFAS may affect estrogen homeostasis and foetal growth during pregnancy, and that estrogens may mediate the relationship between PFAS exposure and foetal growth after examining 424 mother-infant pairs [245].

Some of the studies also explored the linkage between the PFAS exposure and liver function [31,251,252]. In 47,092 adult participants, Gallo et al. (2012) found a positive association between PFOA and PFOS concentrations and serum ALT level. On the other hand, the relationship with bilirubin appeared to increase at low levels of PFOA and decrease at higher levels [246]. In 1002 individuals from Sweden, Salihovic et al. (2018) have also found a positive association of PFHpA, PFOA, PFNA, and PFOS concentrations and ALT activity, but also positive associations of PFHpA, PFOA, PFNA, PFDA, and PFUnDA with ALP. These authors noted that the changes of investigated PFAS concentrations were positively associated with gamma glutamyl transferase (GGT) levels and inversely associated with the changes in circulating bilirubin [30]. On the other hand, in 2883 participants, (1801 non-obese and 1082 obese), Jain and Ducatman investigated the connection between liver function alterations and various PFAS. They concluded that connections might only be observed in the obese participants: alanine aminotransferase (ALT) was positively associated with PFOA, PFHxS, and PFNA. On the other hand, PFOA and PFNA were associated with GGT [240]. Epidemiological studies revealed a connection between PFAS and decrease in vaccination antibody production in early infants and children, especially having in mind that, if breastfed, they have a relatively high exposure and may be more susceptible as their immune system develops. Abraham et al. (2020) found significant associations between the concentration of PFOA, but not PFOS, and adjusted levels of vaccine antibodies against Haemophilus influenza type b, tetanus and diphtheria for which no observed adverse effect concentrations (NOAECs) were 12.2, 16.9 and 16.2 µg/L, respectively. Furthermore, PFOA levels were shown to be inversely related to the interferon gamma (IFN-γ) production of ex-vivo lymphocytes after stimulation with tetanus and diphtheria toxoid [248]. Furthermore, Budtz-Jorgensen E and Grandjean P (2018) found an approximate BMDL of 1 ng/mL serum for both PFOS and PFOA for the serum concentrations of specific IgG antibodies against tetanus and diphtheria at ages 5 and 7 as outcome parameters. These authors proposed the reference concentration of about 0.1 ng/mL as the serum-based target, a level which is below the most reported human serum-PFAS concentrations [249]. Grandjean et al. (2017) discovered that prenatal exposure to PFAS had an inverse relationship with antibody concentrations five years later, while concentrations measured at 3 and 6 months of age had the highest inverse relationships with antibody concentrations at 5 years of age, especially for tetanus [250]. The same authors have found that diphtheria antibody concentrations dropped at higher PFAS concentrations at 13 and 7 years after booster vaccinations at 5 years of age; the correlations were statistically significant for PFDA at 7 years and PFOA at 13 years, implying a 25% decrease for each doubling of exposure [251]. 

## 4. Measures to Mitigate the Impact of PFAS on Human Health and the Environment

### 4.1. Regulatory Measures Aimed to Restrict or Ban the Use of PFAS

In light of the past and current evidence, there is an increasing awareness and a general agreement that PFAS substances need to be regulated at multiple levels to minimize their adverse effects on human health and the environment. From this perspective, a first step towards the regulation of PFAS substances occurred in 2000, when the American company 3M, one of the leading manufacturers of PFOS, announced the phase-out of this substance and its related compounds [252] a decision that was followed six years later by the commitment from the major fluorochemical producers worldwide to strongly reduce the emissions and the product content of PFOA, its precursors and other PFAS substances with longer chain carbons [253]. At the level of the European Union (EU), the Stockholm Convention on Persistent Organic Pollutants (POPs) signed in 2001 to restrict the use of substances with high persistence and bioaccumulative potential, led to the ban of most PFOS uses and related compounds [254]. In 2009, the production and use of PFOS and its precursor PFOSF were also globally restricted [255] while starting from 2015 onwards, PFOA, its salts and hundreds of substances that can act as precursors generating other PFAS species under certain conditions, have been progressively listed in the annex B of Stockholm Convention [256]. These regulatory measures focused on individual PFAS substances have induced the PFAS world manufactures to find alternative substitutes resulting in a progressive shift towards shorter chain molecules such as PFBS, PFHxA or PFHxS supposed to have a safer profile. However, contrary to what initially assumed, increasing evidence support the notion that these alternative molecules still pose a concern for wildlife and humans due to their persistence and high mobility across the different environmental compartments. In addition, the replacement of the so-called legacy PFAS with new alternatives has created a substantial gap through the introduction of other substances devoid of specific regulation at global or national level and long-term ecotoxicological data. In this respect, it is important to mention that approximately a dozen of PFAS are included in the Candidate List of Substances of Very High Concern (SVHC) pursuant to Article 59(10) of REACH Regulation (EC No 1907/2006) satisfying the Annex XIII criteria for Persistent, Bioaccumulative and Toxic (PBT) or very Persistent and very Bioaccumulative (vPvB) substances. Moreover, a limited number of PFAS including PFOS, PFOA, PFNA, PFDA and APFO is currently classified according to the harmonized EU classification and labelling requirements of the CLP Regulation (EC No 1272/2008) and some other compounds such as 6:2 FTOH and PFHpA will undergo harmonized classification in the nextcoming future. However, it is quite obvious that none of these single measures is sufficient to cover the enormous number of PFAS substances currently present on the market and that each regulatory measure targeting individual PFAS will just promote its replacement in favor of other unregulated PFAS, requiring extensive assessment of their impact on humans and wildlife. Based on these considerations and in light of the tremendous efforts that would be required to assess and regulate each individual PFAS, there is an increasing consensus that these molecules need to be regulated as a group of substances through a comprehensive legislative measure adopted at the level of EU. To this extent, while Norway and Germany have respectively presented a restriction proposal for PFHxS and its salts as well as PFHxA and its salts, the European Agency for chemical substances (ECHA), upon the specific request from the European commission (CE), is planning to implement a comprehensive legislative measure targeting the whole category of PFAS [257]. Similarly, governments worldwide are increasingly adopting broad regulatory approaches to target all PFAS substances within categories associated with wide-dispersive uses. In this respect, however, it will be of crucial importance to identify the essential uses of PFAS within those categories and to carefully evaluate risks and benefits of each specific case [258]. Apart from this, legislative measures have been adopted by the European Parliament to protect water resources intended for human consumption as in the case of the EU Directive 2020/2184 which defines that by 2026, levels of PFAS in drinking water shall comply with strict parameters and quality standards set by Member states. Taken together, it is clear that policy makers and governments are becoming increasingly aware that broad measures to regulate PFAS production, uses and emissions are critically needed to protect the human health and the environment.

### 4.2. Remediation Technologies for PFAS Removal

In light of the growing awareness of the risks posed by PFAS on wildlife and humans, a number of treatment technologies has been designed and applied to the different environmental compartments taking advantage of the physicochemical properties of PFAS substances. In line of principle, both on-site and off-site methods can be used to immobilize, transform or destroy PFAS in the environmental matrix of interest, but their huge variability, the increasing number of alternatives and considerations related to costs effectiveness and environmental impact, have narrowed the number of effective treatments to a bunch of PFAS (i.e., PFOS and PFOA) for which the environmental fate, transformation pathways and physicochemical properties are well known [8]. Given the importance of water sources for human health, the vast majority of the efforts have historically focused on the development of effective methods to sequester or remove PFAS substances from contaminated liquid streams such as groundwater, drinking water, wastewater and other industrial effluents while only later the interest has moved towards the implementation of valid strategies for soil remediation [71,259]. Here below, some of the most common remediation techniques for PFAS remediation will be briefly presented while the reader interested to a more detailed description is referred to other excellent reviews [8,73].

#### 4.2.1. Remediation Technologies for Treatment of PFAS-Contaminated Water

The need of protecting drinking water sources from PFAS contamination has prompted researchers to develop a number of different approaches that have been assessed at the bench and field scale level. However, very soon it was realized that the surfactant properties and the stability of PFAS substances rendered those methods relying on PFAS volatilization (such as air sparging or biosparging) largely ineffective and those relying on PFAS degradation (such as chemical oxidation/reduction or thermal destruction) effective only under harsh operating conditions. For this reason and in consideration of their full-scale applicability, their proven efficiency and their cost-effective profile, some techniques have emerged becoming the main choice for water streams treatment. Among them, the adsorption processes based on ion exchange resins (IER) or granular activated carbon (GAC) as well as filtration technologies such as reverse osmosis (RO) and nanofiltration (NF) are the most common [8].

PFAS removal by IER exploits the amphiphilic nature of these substances and involves both electrostatic interactions between the positively charged functional groups of the resin polymers and the negatively charged head of PFAS species (such as PFOA and PFOS) in the appropriate pH range, but also hydrophobic interactions between the non-polar carbon chain tail and the resins backbone. As a result, PFAS are transferred from the aqueous phase to a solid matrix with high selectivity and efficiency [260,261]. Both single-use and renewable resins have been employed, the former being more suitable to treat low PFAS concentrations typically found in potable water systems until a breakthrough occurs, while the latter is more indicated to treat streams with high PFAS content. This method has shown to be effective in removing a broad range of PFAS existing at concentrations from part per trillion (ppt) to hundreds of parts per billion (ppb) in water streams of influent and effluent origin [262,263,264]. A similar mechanism of hydrophobic interactions drives PFAS removal through GAC adsorption, which is the most frequently used method for water decontamination by PFAS. Evidence from the literature indicate that GAC can proficiently remove long chain PFAS (such as PFOS, PFOA and PFNA) in the range of ppt or ppb until breakthrough starts to occur, while is less effective in case of short chain PFAS which are generally characterized by lower GAC loading capacities and faster breakthrough [265,266,267,268]. Potential drawbacks of this technique are represented by the need of regenerating the active carbon with a certain frequency and the co-occurrence of other organic contaminants that might decrease the overall removal efficiency. Advanced filtration methods such as RO and NF are energy-effective techniques that rely on a diffusion process through which pressurized water streams are forced through a polymer-made semipermeable membrane. The permeate treated water is thus decontaminated while the PFAS are retained and concentrated, requiring subsequent disposal or treatment [269]. Both RO and NF alone or in combination have been shown to remove PFOS with efficiencies greater than 90–99% over a wide range of concentration (0.5–1500 mg/L) [270,271], with a good applicability to long chain and short chain PFAS [262,272], but the costs associated with their use might become a limiting factor. In addition to these techniques, other methods have been tested to treat PFAS contaminated water streams, although some of them still need to be assessed on a field-scale level or require harsh conditions and high doses of other reagents. Some examples are represented by electrochemical oxidation (with ozone, ammonium persulfate or hydrogen peroxide/Fenton reagent) [273,274,275], plasma treatment (high voltage electrical discharges generating free radical species) [276], sonolysis (mineralization through high-frequency ultrasound) [277] and foam fractionation (sequestration of PFAS into air or ozone bubbles formed at the air-water interface) [278].

#### 4.2.2. Remediation Technologies for Treatment of PFAS-Contaminated Soil

The amphipathic properties of PFAS substances are largely responsible of PFAS adsorption to soil particles and sediments, a process which is influenced by the environmental pH, soil geochemical composition, the total organic carbon content (mainly humic and fulvic acids), the presence of proteins or saccharides as well as the content of iron and aluminum oxides [71]. Several remediation techniques have been developed and tested to immobilize, remove or destroy PFAS substances in the soil while others are still under investigation and full implementation from the laboratory to the field scale size. These include immobilization, soil washing/flushing, thermal destruction/desorption, reduction/oxidation, ball milling and bioremediation [279]. The immobilization technique is based on the addition of minerals (such as modified clay or activated carbon) or stabilization agents (such as Portland cement) to the soil, which absorb PFAS or create an impermeable layer, thus limiting their diffusion into groundwater or interstitial soil water [280]. For example the efficacy of colloidal activated carbon has been shown to positively correlate with the soil clay content but negatively with the total organic carbon content, resulting in an absorption efficiency of 81% for PFOA, 85% for 6:2 FTSA and 86% for PFHxS, under optimal conditions [281]. More recently, several additives including powdered activated carbon (PAC) and modified clay (Rembind^®®^) have been tested in soils spiked with PFAS compounds of variable chain lengths and chemical nature in a bench scale setting. Here, it was found that the leachability of 13 out of 14 PFAS (with the sole exception of PFBA) significantly decreased after addition of PAC (average value 70%) and Rembind^®®^ (average value 94%). Of note, the leachability of long chain PFAS such as PFOA, PFOS, PFNA, PFDA, PFHxS, 6:2 FTSA, 8:2 FTSA, and FOSA decreased up to 2–3 orders of magnitude (>99.0–99.9%) indicating a promising efficacy for future field application [280]. Soil washing requires the excavation of soil, the removal of larger particle sizes and the subsequent treatment of the finest ones with an extracting agent [282]. By contrast, soil flushing does not require mobilization of materials but rather the injection of the extraction fluid on-site and its subsequent recovery [283]. Some highly water-soluble PFAS such as PFOS, PFOA or PFHxS are amenable to water solubilization while other methods such as ultrasounds, the use of organic solvents and amendments such as chelating agents, acids or surfactants might be necessary to extract other PFAS substances with higher hydrophobicity [284,285,286,287]. Two studies conducted on PFOS have shown removal efficiencies greater than 99%, with slight lower decreases in sandy clay soils (94% in comparison to 99% removal in presence of sandy soils) [279]. Potential drawbacks are represented by the mobilization of large volumes of soil and the need of other treatment methods, beside significant costs for a full-scale implementation and extensive time for remediation. Processes that ultimately cause PFAS destruction have also been developed. Among them, thermal destruction is an energy-intensive process based on the application of heat with temperatures ranging from 350 to 900 °C leading to the PFAS desorption from soil matter and the formation of a gas stream, which is then subdued to further heating (>1200 °C) to induce PFAS breakdown and retention of the fluoride atoms in a scrubber [279,288]. While initially characterized by unsatisfactory efficiency due to technical limitations in the delivery of the necessary heat in a uniform and cost-effective way, this technique has been progressively improved as assessed by a recent bench-scale study wherein soil samples contaminated with a mixture of 9 different PFAS were subdued to gradual heating increases. Here, 350 °C were found to be sufficient to remove >99% of PFCAs and FOSA while 450 °C were necessary to achieve the same extent of PFSAs (PFHxS, PFOS) removal. At 550 °C the removal of PFPeA was >97%, but other PFCAs were only removed to 71–93%, revealing a positive correlation between the entity of applied heat and the removal efficiency with the exception of shorter chain PFAS such as PFBA that instead exhibited a negative trend removal in the range of 150–400 °C [289]. Despite the technical improvements, thermal destruction/desorption is an expensive method with a significant environmental impact which also requires substantial costs for the infrastructure. As previously noted, chemical reduction/oxidation is based on the addition of highly reactive chemicals on the contaminated plume either on-site or off-site. In the former case the reagents are introduced through vertical injection wells located upstream the contamination (and coupled to extraction wells downstream if it is desired to protect groundwater) while in the latter the soil needs to be adequately mixed to favor the homogenous dispersion of the reagents. In this respect, earlier reports showed that heat activated persulfate was able to completely degrade PFOA at moderate temperature (20–40 °C) but under very low pH conditions [290]. In more environmentally-relevant conditions iron-activated persulfate was recently found to induce around 64% degradation of PFOA under illuminated anoxic conditions and at room temperature [291]. Other studies have focused on remediation of soil contaminated with AFFFs. In a first report, Briton et al. conducted batch experiments on groundwater and aquifer sediments spiked with two different AFFs formulations to assess the removal efficacy of heat activated persulfate. Here, heat-activated persulfate under acidic conditions led to the conversion of fluorotelomer-based and sulfonamide-based PFAA precursors into PFCAs, which were eventually mineralized, with a marked effect on 6:2 FtTAoS, consistently with other findings from the same authors [292]. By contrast, the treatment was poorly effective against other PFSAs (i.e., PFOS and PFHxS) revealing also the impossibility of mass balance determination of fluorine content. The authors concluded that heat-activated persulfate might be useful to remediate soil contaminated by AFFFs containing PFCAs or fluorotelomer-based PFAS but not other types [293]. The remediation with heat activated persulfate appears to be much more problematic in case of PFOS since poor [294] or no defluorination has been observed even under acidic conditions and high persulfate doses [274,292]. In general, the use of heat activated persulfate has some important drawbacks including the generation of undesired byproducts, including short chain PFCAs and the mobilization of heavy metals into the aquifer due to its acidification. Thus, major improvements in PFOS removal might derive from the use of reductive processes as evidenced by recent studies wherein UV-generated solvated electrons were found to effectively induce PFOS reductive defluorination (while generating shorter chain perfluorinated acids) without the use of chemicals [295], a method that has shown promising results also when applied to PFOS containing AFFFs [296]. Another technique causing the mechanochemical destruction of PFAS is the ball milling wherein the contaminated soil is placed into specific reactors and forced to collide with solid balls causing chemical transformations and physical grinding [297]. In this respect, near complete removal of PFOS and PFOA were reported in earlier investigations using a planetary ball milling and KOH as co-milling agent [298], while a more recent study reported a decrease in PFOS concentrations up to 99% in amended sand and 96% in site soils from a Canadian firefighting training area [298]. The authors concluded that ball milling might represent a promising, energy-effective and cost-effective method to remediate soil contaminated by PFAS. Lastly, another promising technique with high environmental sustainability and low operational costs is represented by the bioremediation that takes advantage of microorganisms (i.e., bacteria and fungi) or plant species to respectively promote PFAS transformation/degradation and their uptake/stabilization. While historically PFAS substances were shown to be recalcitrant to biodegradation by microorganisms, some exceptions have been recently identified. For instance, the *Pseudomonas aeruginosa* strain HJ4 grown in activated sludge and soil samples from a WWTP in Korea, was found to induce 67% degradation of PFOS after 48 h of incubation [299], while the YAB1 strain from *Pseudomonas parafulva* was seen to biodegrade up to 32% of PFOA in soil samples from a PFC production plant in China [300]. Later studies provided evidence that the strain *Pseudomonas plecoglossicida* 2.4-D was able to use PFOS as a carbon source when cultured in liquid medium and to actively degrade up to 75% of PFOS present at levels of 0.5% *w*/*w* in contaminated soil samples after six months [301]. More recently, two strains of *Pseudomonas* (PS27 and PDMF10) isolated from PFAS-contaminated sites were found to bioaccumulate (but not degrade) and thus to remove 32% and 28% of PFHxS contamination within 10 days, respectively [302]. An alternative bioremediation strategy is represented by the use of plants that bioaccumulate PFAS substances in their root or leaves leading to the removal of these contaminants from the soil without inducing their degradation [71]. In a former report from Gobelius et al., analyzed the uptake of 26 PFAS in different plant species grown on the soil near to a fire training site at Stockholm Arlanda airport, contaminated with levels of total PFAS ranging from 16 to 160 ng/g dw. Among the others, the highest PFAS content was seen in the leaves of silver birch (*Betula pendula*) and the needles of Norway spruce (*Picea abies*), with concentrations as high as 97 ng/g ww and 94 ng/g ww [201]. More recently, Huff et al. conducted a greenhouse study wherein they assessed the capacity of eight herbaceous and seven woody plants to absorb PFPeA, PFHxA, PFOA, PFBS, PFHxS, and PFOS added weekly to the irrigation water. Here, species-specific differences were found in tissue concentrations of individual PFAS and their accumulation pattern but the short chain PFPeA exhibited the highest uptake while PFOS the lowest. *Festuca rubra* was the most effective species and was able to hyperaccumulate all six PFAS compounds tested, showing a recovery of the PFPeA, PFHxA, and PFBS applied in the above-ground biomass greater than 25%. Of note, *Equisetum hyemale*, *Schedonorus arundinaceus* and *Amaranthus tricolor*, were the other herbaceous species showing above-average accumulation of multiple PFAS compounds [303]. Taken together, these data demonstrate that there is growing awareness on the impact of PFAS substances in the environmental media and the need of developing effective remediation strategies with elevated sustainability in terms of cost-effectiveness and environmental footprint as well as wider applicability to full-scale contexts. While it is plausible that the continuous technological advances will make this ambitious goal achievable in the next future, many efforts should be made in the short term to implement promising approaches from a laboratory to a field scale. Importantly, significant improvements might derive from the suitable combination of different remediation techniques by designing the most appropriate treatment train approaches, which are expected to represent the new frontier of PFAS removal from contaminated environmental media.

## 5. Concluding Remarks

Since their introduction, PFAS substances have provided some advantages to our society over the last 60 years, but in the same time have also raised major concerns for human health and the environment. Compared to this long temporal frame, the scientific knowledge related to PFAS behavior and effects in living organisms and humans has emerged quite recently and has been focused on a number of few PFAS substances such as PFOA or PFOS. The extremely high number of PFAS substances which currently exceeds 4700, renders almost impossible to assess the specific effects that each single compound might cause in the ecosystems and human subpopulation over the short and long term. Similarly, it is becoming increasingly clear that any regulatory measure will not be effective as long as it will be targeting individual PFAS substances, rather than the whole group. An additional layer of complexity is given by the fact that even the phase out of PFAS substances with a recognized adverse impact on wildlife and human health, or the design of supposedly safer alternatives appears to be not sufficient to effectively control the risks associated with these substances and would rather delay the adoption of the appropriate legislative actions. In light of the extreme environmental persistence of PFAS, their ubiquitous distribution and the absence of adequate testing procedures or models able to fully describe their long-term effects, a key aspect relates to the application of the precautionary principle as driver of any future decision. In keeping with these considerations, wider legislative measures have been provisionally planned (i.e., global restriction of PFAS molecules) by the ECHA and some member states to regulate PFAS at the level of EU. In the same time, significant advances are expected to emerge from the identification of the most effective remediation technologies to remove PFAS in the environmental media in a cost-effective and environmentally sustainable way. In this regard, bioremediation techniques and the combined use of different treatment train approaches represent promising strategies which are intensively investigated. In conclusion, PFAS pose a number of important challenges to our society that has to reconcile the need of maintaining competitiveness of the European industry, promote technical innovation and ensure the highest protection level to the environment and human health for the future generations. Taking into account these aspects, it is very likely that PFAS management will be one of the greatest tasks of our time and a matter of intense scientific as well as political debate in the next coming years.

## Figures and Tables

**Table 1:** List of main PFAS categories and subgroups.

Non-Polymeric PFAS			
Perfluorinated PFAS		Polyfluorinated PFAS	
Subgroup	Examples	Subgroup	Examples
Perfluoroalkyl acids (PFAAs) Perfluoroalkane sulfonic acids & sulfonates (PFSAs) Perfluoroalkane sulfnic acids (PFSIAs) Perfluorocarboxylic acids & carboxylates (PFCAs) Perfluoroalkyl phosphonic acids (PFPAs) Perfluoroalkyl phosphinic acids (PFPIAs)	PFBS, PFHxS, PFOS PFOSI PFBA, PFHxA, PFOA C8-PFPA C8/C8-PFPiA	Fluorotelomer compounds (FT)	6:2 FTO, 8:2 FTI
		Perfluoroalkane sulfonamido compounds (Me/Et/Bu-FASAs) Miscellaneous	MeFOSA, FOSE 4,8-Dioxa-3H-perfluorononanoate
Perfluoroalkyl ether acids (PFEAs)	GenX, Adona, F-53B		
Perfluoroalkane sulfonamides (FASA)	FOSA		
Perfluoroalkane sulfonyl fluorides (PASFs)	PBSF, POSF		
Perfluoroalkyl iodides (PFAIs)	PFHxI		
Perfluoroalkanoyl fluorides (PAFs)	POF		
Perfluoroalkyl aldehydes (PFALs)	PFNAL		
**Polymeric PFAS**			
**Subgroup**	**Examples**		
Fluoropolymers	PVDF, FEP, PFA, ETFE, PTFE (Teflon)		
Side-chain Fluorinated Polymers	Fluorinated urethane/acrylate/methacrylate/oxetane plolymers		
Perfluoropolyethers (PFPEs)	PFPE-BP, Fluorolink-PFPE		

Classification of PFAS family substances adapted from Buck et al., 2011 [1] and OECD, 2013 (OECD/UNEP Global PFC Group, Synthesis paper on per- and polyfluorinated chemicals, PFCs, Environment, Health and Safety Directorate, OECD. Paris, 2013) [57].

**Table 2:** List of typical PFAS uses in industrial and consumers products.

SECTOR OF USE	TYPE OF USE
Non-Polymeric PFAS	
Fire prevention	Fire-fighting foams such as foams based on aqueous films (Acqueous Film-Forming Foams, AFFs)
Biocides	Active products in plants grow regulators (PGRs) Active or inert (emulsifiers, solvents, carriers, aerosol propellants) ingredients in pesticides
Electronic	Flame retardants
Aviation and Aerospace	Additives for hydraulic fluids
Metal plating	Humectants and anti-fog agents
Household Products	Surfactants in floor cleaning; treatment for textiles, leather, carpets; car waxes
Building and Construction	Additives in coatings and paints
Medical Products	Stain-resistant and water-repellent articles, X-ray film
Personal care products	Cosmetics, makeup, nail polish, shampoo
Metal plating	Wetting agent, anti-mist agents
Oil and mining production	Surfactants used in oil-well production and mining flotation
PFAS synthesis	Use as monomers for the synthesis of fluoropolymers with fluorinated side chain
Automotive	Treatment for external surfaces and internal leather coatings, textiles or carpets
Textiles and leather	Treatment aimed to create a coating with oil-water-stain-repellent properties
Semiconductors	Use in the production of semiconductor chips
**Polymeric PFAS**	
Fire prevention	Raw materials for firefighting equipment, protective clothes and fuel repellents
Electronic	Insulators and materials for welding
Aviation and Aerospace	Insulators, sleeves
Household Products	Non-stick coatings
Building and Construction	Coating of architectural materials, additives in paints, dyes, stains and sealants
Medical Products	Use in surgical patches, biocompatible human implants and medical prosthesis
Personal care products	Use in dental floss and lotions
Oil and mining production	Use in lining of gas pipes
Automotive	Mechanical components, seals and lubricants
Textiles, leather and clothing	Use in the manufacture of clothing and housewares as well as in coatings having oil-water-repellent properties
Semiconductors	Use as fluids in mechanical vacuum pumps
Energy	Film for solar panels
Paper and packaging	Use in water-oil-repellent materials, paperboard, and bags for food packaging
Cables and wiring	Coatings resistant to weathering, flame and soil
Food processing	Production of materials used for cooking (non-stick pans) and food storage (containers)

**Table 3:** List of PFAS extended chemical names and acronyms.

PFAS Chemical Name	PFAS Acronym	PFAS Chemical Name	PFAS Acronym
Perfluorooctanoic acid	PFOA	N-ethyl perfluorooctane sulfonamide	Et-FOSA
Perfluorooctane sulfonic acid	PFOS	N-methyl perfluorooctane sulfonamide	Me-FOSA
Perfluorooctanesulfonamide	PFOSA/FOSA	N-ethyl-perfluorooctane sulfonamido acetic acid	N-Et-FOSAA
Perfluorooctane sulfinic acid	PFOSI	N-methyl-perfluorooctane sulfonamido acetic acid	N-Me-FOSAA
Perfluorononanoic acid	PFNA	2-(N-Methyl-perfluorooctane sulfonamido) acetic acid	Me-FOSAA/Me-PFOSA-AcOH)
Perfluorononanal	PFNAL	2-(N-Ethyl-perfluorooctane sulfonamido) acetic acid	Et-FOSAA/Et-PFOSA-AcOH
Perfluorononane sulfonic acid	PFNS	perfluorooctane sulfonamido ethanol	FOSE
Perfluoroundecanoic acid	PFUnDA	N-ethyl perfluorooctane sulfonamido ethanol	Et-FOSE
Perfluoroundecanoate	PFUnA	perfluorohexane sulfonamide	FHxSA
Perfluorodecanoic acid	PFDA	bis(perfluorooctyl)phosphinic acid	C8/C8-PFPiA
Perfluorododecanoic acid	PFDoA	6:2 Fluorotelomer olefin	6:2 FTO
Perfluorodecane sulfonic acid	PFDS	6:2,8:2,10:2 fluorotelomer alcohol	6:2,8:2,10:2 FTOH
Perfluorobutanoic acid	PFBA	6:2,8:2 fluorotelomer sulfonic acid	6:2,8:2 FTSA
Perfluorobutane sulfonic acid	PFBS	6:2 fluorotelomer thioether amido sulfonate	6:2 FtTAoS
Perfluorophosphonic acid	PFPA	8:2 fluorotelomer iodide	8:2 FTI
Perfluoropentanoic acid	PFPeA	8:2 fluorotelomer unsaturated carboxylic acid	8:2 FTUCA
Perfluoropolyether-benzophenone	PFPE-BP	9-chlorohexadecafluoro-3-oxanone-1-sulfonic acid	6:2 Cl-PFESA (F-53B)
Perfluorohexanoic acid	PFHxA	1-chloroeicosafluoro-3-oxaundecane-1-sulfonic acid	8:2 Cl-PFESA
Perfluorohexyl iodide	PFHxI	Polyvinylidene fluoride	PVDF
Perfluorohexane sulfonic acid	PFHxS	Fluorinated ethylene propylene	FEP
Perfluoroheptanoic acid	PFHpA	Perfluoroalkoxy polymer	PFA
Perfluoroheptane sulfonic acid	PFHpS	Ethylene tetrafluoroethylene	ETFE
Perfluorotridecanoic acid	PFTrDA	Polytetrafluoroethylene	PTFE
Perfluorooctanoyl fluoride	POF	Hexafluoropropylene oxide dimer acid	HFPO-DA/GenX
Perfluorobutane sulfonyl fluoride	PBSF	3H-perfluoro-3-[(3-methoxy-propoxy)] propanoic acid	ADONA
Perfluorooctane sulfonyl fluoride	POSF	Ammonium pentadecafluorooctanoate	APFO
Ammonium perfluoro(2-methyl-3-oxahexanoate)	PMOH		

**Table 4:** Selected in vitro studies (published since 2010) exploring the toxicity of polyfluoroalkyl substances (PFAS).

Cell Type	Substance	Treatment Concentration	Incubation Time	Effects	Ref.
thyroid follicular cells	PFOS PFOA	PFOS or PFOA (1–100 mM)	Cytotoxicity: 1 h	PFOS, but not PFOA, acutely and reversibly inhibited iodide accumulation by FRTL-5 thyrocytes, as well as by HEK-293 cells transiently expressing the Sodium Iodide Symporter (NIS) PFOS prevented NIS-mediated iodide uptake and reduced intracellular iodide concentration in iodide-containing cells, mimicking the effect of the NIS inhibitor perchlorate PFOS did not affect iodide efflux from thyroid cells	(Conti, Strazzeri, and Rhoden 2020) [213]
FTC-238/hrTPO/RSK008 cells	PFOS PFOA	10^−9^, 10^−8^, 10^−7^, 10^−6^, 10^−5^, 10^−4^ M	/	Decreased TPO activity	(Song et al., 2012) [214]
rat thyroid line-5 (FRTL-5)	PFOS PFOA	1, 10, 10^2^, 10^3^, 10^4^, and 10^5^ nM	72 h	At concentration of 10^5^ nM PFOA/PFOS, a significant inhibition of cell proliferation, mainly due to increased cell death, was found PFOA and PFOS enter thyroid cells by a gradient-based passive diffusion mechanism	(Coperchini et al., 2015) [215]
rat thyroid line-5 (FRTL-5)	FOA, PFOS, perfluorobutanesulfonic acid (PFBS), perfluorobutanoic acid (PFBA), pentafluoropropionic anhydride (PFPA), perfluoropentanoic acid (PFPeA)	0.0001; 0.001; 0.01; 0.1; 1; 100 μM	24 h	Neither long nor short-chain PFCs affected cell viability (apart from PFOS 100 μM), or interfered with cAMP production Short-chain PFCs have no acute cytotoxic effect on thyroid cells in vitro	(Croce et al. 2019) [216]
Human hepatoma cell line (HepG2)	perfluorohexane sulfonate (PFHxS), perfluorooctane sulfonic acid (PFOS), perfluoroctanoic acid (PFOA), perfluorononanoate (PFNA), perfluorodecanoate (PFDA), perfluoroundecanoate (PFUnA), and perfluorododecanoate (PFDoA).	2 × 10^−7^, 1 × 10^−6^, 2 × 10^−6^, 1 × 10^−5^, 2 × 10^−5^ M	24 h	Except for PFDoA, all the other PFAS increased ROS generation For PFHxS and PFUnA the observed ROS increases were dose-dependent Cells exposed to PFOA were found to have a significant lower total antioxidant capacity (TAC) compared with the solvent control, whereas a non-significant trend in TAC decrease was observed for PFOS and PFDoA and an increase tendency for PFHxS, PFNA and PFUnA	(Wielsøe et al., 2015) [218]
Human Embryo Liver L-02 Cells	PFOS	0, 50, 100, 150, 200, or 250 μmol/L	24 or 48 h	Decreased cell activities, enhanced ROS levels in a concentration-dependent manner Decreased mitochondrial membrane potential (MMP), and induced autophagy and apoptosis Enhanced expression of Bax, cleaved-caspase-3, and LC3-II Induced autophagy; decreased MMP; and lowered Bcl-2, p62, and Bcl-2/Bax ratio ROS-triggered autophagy is involved in PFOS-induced apoptosis in L-02 cells	(Zeng et al., 2021) [219]
Human HepaRG liver cells	PFOA, PFOS, and perfluorononanoic acid (PFNA)	6.25, 12.5, 25, 50, 100, 200, 400 μM	6, 24, or 72 h	All PFAS induced an increase in cellular triglyceride levels, but had no effect on cholesterol levels PFOA, PFOS, and PFNA increase triglyceride levels and inhibit cholesterogenic gene expression PFAS induce endoplasmic reticulum stress, which may be an important mechanism underlying some of the toxic effects of these chemicals	(Louisse et al., 2020) [220]
HepaRG cell line	PFOS PFOA	100, 250, 500, 750 μM PFOA 50, 100, 250, 500 μM PFOS	/	Cholesterol levels in HepaRG cells were not affected by PFOA or PFOS Both substances strongly decreased synthesis of a number of bile acids The expression of numerous genes whose products are involved in synthesis, metabolism and transport of cholesterol and bile acids was strongly affected by PFOA and PFOS at concentrations above 10 µM Both substances led to a strong decrease of CYP7A1, the key enzyme catalyzing the rate-limiting step in the synthesis of bile acids from cholesterol, both at the protein level and at the level of gene expression Both substances led to a dilatation of bile canaliculi	Behr et al., 2021) [221]
Neurons	PFOS PFOA	30–300 µM	30 min	Both PFOS and PFOA can accumulate in cultured neurons and elevate calcium concentrations via release of intracellular calcium stores 1,4,5-trisphosphate receptors (IP3Rs) and ryanodine receptors (RyRs) were found to take part in PFOS or PFOA inducing calcium release from calcium stores Calcium release from intracellular stores may partially account for the perturbation of calcium homeostasis caused by PFOS or PFOA	(Liu et al., 2011) [222]
Primary rat cortical cultures and hiPSC-derived neuronal co-cultures	PFOS PFOA	0.01, 0.1, 1, 10, 100 µM	/	PFOS and PFOA inhibited the GABA-evoked current and acted as non-competitive human GABAA receptor antagonists Network activity of rat primary cortical cultures increased following exposure to PFOS (LOEC 100 µM)	(Tukker et al., 2020) [223]
Rat primary hippocampal neurons and astrocytes	PFOS	25, 50, 75, 100, 125 μM for neurons 15, 25, 50, 75, 100 μM for astrocytes	24 h	Redox imbalance, increased apoptosis and abnormal autophagy in rat primary hippocampal neurons. In astrocytes: altered extracellular glutamate and glutamine concentrations, decreased glutamine synthase activity, as well as decreased gene expression of glutamine synthase, glutamate transporters and glutamine transporters in the glutamate-glutamine cycle	(Li et al., 2017) [224]
primary rat embryonic neural stem cells (NSCs)	PFOS	12.5–100 nM	48 h	Increase in neuronal differentiation Increased number of CNPase-positive cells, pointing to facilitation of oligodendrocytic differentiation Upregulation of PPARγ with no changes in PPARα or PPARδ genes Upregulated mitochondrial uncoupling protein 2 (UCP2) Induced Ca^2+^ activity	(Wan Ibrahim et al., 2013) [225]
rat primary neurons and neural stem cells (NSC)	PFOS PFOA	1–250 μM	24 h	No effects on cell viability or proliferation in primary neurons PFOS exposure increased the NSC proliferation at the lowest concentration tested (1–100 μM) PFOS and PFOA caused morphological alterations of NSC-derived neurons. Exposure to 1 and 10 μM PFOA also affected the neurite network and caused an increase in the number of processes and branches per cell NSC, mimicking the immature brain, is clearly more susceptible to PFOS and PFOA exposure than the primary neurons	(Pierozan and Karlsson 2021) [226]
fetal rat testes or seminiferous tubule segments (stage VII-VIII) of adult rats	PFOA	0–100 μg/mL	24 h	Levels of cAMP, progesterone, testosterone and expression of StAR decreased significantly in PFOA 50 and 100 μg/mL. PFOA affected cell populations significantly by decreasing the amount of diploid, proliferating, meiotic I and G2/M phase cells in adult rat testis PFOA did not affect fetal, proliferating or adult rat Sertoli cells but an increased tendency of apoptosis in fetal Leydig cells was observed	(Eggert et al., 2019) [227]
human cell lines such as MCF-7, H295R, LNCaP and MDA-kb2	PFOA, PFOS, and of six substitutes including perfluorohexanesulfonic acid (PFHxS), perfluorobutanesulfonic acid (PFBS), perfluorohexanoic acid (PFHxA), perfluorobutanoic acid (PFBA), ammonium perfluoro(2-methyl-3-oxahexanoate) (PMOH), and 3H-perfluoro-3-[(3-methoxypropoxy) propanoic acid] (PMPP)	various concentrations	24 h when cytotoxicity was assayed in HEK293T, LNCaP or MDA-kb2 cells, for 6 d in MCF-7 cells and for 48 h in H295R cells	PFOA, PFOS and PMOH enhanced 17β-estradiol-stimulated estrogen receptor β activity, and PFOS, PMOH, PFHxA and PFBA enhanced dihydrotestosterone-stimulated androgen receptor activity PFOA and PFOS slightly enhanced estrone secretion, and progesterone secretion was marginally increased by PFOA. All these effects were only observed at concentrations above 10 μM	(Behr et al.,2018) [228]

**Table 5:** Selected in vivo studies (published since 2010) exploring the toxicity of polyfluoroalkyl substances (PFAS).

Species	Substance	Dose and Route of Exposure	Exposure Time	Effects	Ref.
Rats	PFOS	20 or 100 ppm, dietary exposure	7 days	Changes in liver parameters (increased liver weight; decreased plasma cholesterol, alanine aminotransferase, and triglycerides; decreased liver DNA concentration and increased hepatocellular cytosolic CYP450 concentration; increased liver activity of acyl CoA oxidase, CYP4A, CYP2B, and CYP3A; increased liver proliferative index and decreased liver apoptotic index; decreased hepatocellular glycogen-induced vacuoles; increased centrilobular hepatocellular hypertrophy. Thyroid parameters (histology, apoptosis, and proliferation) unaffected.	(Elcombe et al., 2012) [229]
Mice	PFOS	10 mg PFOS/kg b.w./day), oral gavage	14 days	Dysregulated proteins in lipid and xenobiotic metabolism in liver 16 overexpressed glycoproteins associated with neutrophil degranulation, cellular responses to stress, and protein processing in the endoplasmic reticulum (ER)	(D. Li et al., 2021) [230]
Mice	PFOS	100 μg/kg b.w./day and 1000 μg/kg b.w./day, oral gavage	2 months	PFOS accumulated in liver, lungs, kidneys, spleen, heart and brain Its accumulation caused damage in the liver and in the marginal area of the heart PFOS mainly affected glycerophospholipid metabolism and sphingolipid metabolism in liver Up-regulated ceramide and lysophosphatidylcholine (LPC) might lead to liver cell apoptosis Decrease in liver triglyceride (TG) content might result in insufficient energy and cause liver morphological damage	(X. Li et al., 2021) [231]
Rats	PFOA	5 mg/kg b.w./day, oral gavage	28 days	Increase in hepatic (GGT, ALT, AST and ALP) and renal function (urea and creatinine) biomarkers of toxicities Decrease in the activity of the enzymatic antioxidants (CAT, GPx, SOD) in liver and kidney tissue Increase in lipid peroxidation and proinflammatory cytokine IL-1β Decrease of the antiinflammatory cytokine, IL-10	(Owumi, Bello, and Oyelere 2021) [232]
Mice	PFOA	1, 5, 10, or 20 mg/kg/day, oral gavage	10 days	Increase in Dnmt1 with decreased Rasal1 expression at higher levels of PFOA exposure. Rasal1 hypermethylation, followed by the increase in Hdac1, 3 and 4. Increased mRNA expression levels of TGF-β and α-SMA	(Rashid et al., 2020) [233]
Mice	PFHxS	Up to 3 mg/kg b.w./day, oral gavage	Administered before mating, for at least 42 days in F0 males, and for F0 females, through gestation and lactation. F1 pups-directly for 14 days after weaning	Adaptive hepatocellular hypertrophy, concomitant decreased serum cholesterol and increased alkaline phosphatase (S. Chang et al., 2018).	(Chang et al., 2018) [234]
Rats	PFHxS	0.05, 5 or 25 mg/kg b.w./day, oral gavage	From gestation day 7 through to postnatal day 22	PFHxS lowered thyroid hormone levels in both dams and of spring in a dose-dependent manner PFHxS did not change TSH levels, weight, histology, or expression of marker genes of the thyroid gland	(Ramhøj et al., 2020) [235]
Mice		6.1, and 9.1 mg/kg b.w., oral gavage	Neonatal exposure from postnatal day 10	PFHxS induces persistent developmental neurotoxicity and GAP-43 and CaMKII downregulation via the NMDA receptor-mediated PKCs (α and δ)-ERK/AMPK pathways Significant memory impairment in adult mice	(Sim and Lee, 2022) [236]

**Table 6:** Selected human studies (published since 2010) exploring the toxicity of polyfluoroalkyl substances (PFAS).

**Substance**	Population	**Measured Parameters**	**Results**	**Ref.**
PFOS PFOA	middle-aged Danish population; 753 individuals (663 men and 90 women), 50–65 years of age, nested within a Danish cohort of 57,053 participants	serum levels of total cholesterol	Statistically significant positive associations between PFOS, PFAS and total cholesterol level Sex and prevalent diabetes modified the association between PFOA and PFOS and cholesterol	(Eriksen et al., 2013) [237]
PFOS PFOA	815 participants ≤18 years of age from the National Health and Nutrition Examination Survey 1999–2008	dyslipidemia: total cholesterol >170 mg/dL, low-density lipoprotein cholesterol (LDL-C) >110 mg/dL, high-density lipoprotein cholesterol (HDL-C) <40 mg/dL or triglycerides >150 mg/dL.	Serum PFOA and PFOS-positively associated with high total cholesterol and LDL-C, independent of age, sex, race-ethnicity, body mass index, annual household income, physical activity and serum cotinine levels PFOA and PFOS-not significantly associated with abnormal HDL-C and triglyceride levels.	(Geiger et al., 2014) [238]
PFOS PFOA	290 individuals (144 men + 146 women) exposed to background levels of PFOS and elevated concentrations of PFOA through drinking water, aged between 20 and 60 years	expression of genes involved in cholesterol metabolism	Inverse associations between serum PFOA levels and the whole blood expression level of genes involved in cholesterol transport (NR1H2, NPC1 and ABCG1) A positive association between PFOS and a transcript involved in cholesterol mobilisation (NCEH1), and a negative relationship with a transcript involved in cholesterol transport (NR1H3) Reductions in the levels of mRNAs involved in cholesterol transport were seen with PFOA in men (NPC1, ABCG1, and PPARA) and in women (NR1H2 expression) Increase in the levels of a cholesterol mobilisation transcript (NCEH1) in women. PFOS was positively associated with expression of genes involved in both cholesterol mobilisation and transport in women (NCEH1 and PPARA)	(Fletcher et al., 2013) [239]
PFOA PFOS PFHxS PFNA PFDA	2883 participants, (1801 non-obese and 1082 obese), aged more than or equal to 20 years old	liver function parameters: AST, ALT, GGT, ALP, and total bilirubin (TB)	Among obese participants only, alanine aminotransferase (ALT)-positively associated with PFOA, PFHxS, and PFNA PFOA and PFNA were associated with gamma GGT in obese participants	(Jain and Ducatman 2019) [240]
14 PFCs	Healthy men from the general population, median age of 19 years	total testosterone (T), estradiol (E), sex hormone-binding globulin (SHBG), luteinizing hormone (LH), follicle-stimulating hormone (FSH) and inhibin-B and Semen samples analysis	PFOS levels-negatively associated with testosterone, calculated free testosterone (FT), free androgen index (FAI) and ratios of T/LH, FAI/LH and FT/LH Other PFCs were found at lower levels than PFOS and did not exhibit the same associations. PFC levels were not significantly associated with semen quality	(Joensen et al., 2013) [241]
PFOA PFOS PFHxS PFNA	1682 males and females 12 to 80 years of age	testosterone (T), thyroid stimulating hormone (TSH), and free and total triiodothyronine (FT3, TT3) and thyroxine (FT4, TT4)	Exposure to PFAS may be associated with increases in FT3, TT3, and FT4 among adult females During adolescence, PFAS may be related to increases in TSH among males and decreases in TSH among females No significant relationships were observed between PFAS and T in any of the models	(Lewis, Johns, and Meeker 2015) [242]
PFOS PFOA	3076 boys and 2931 girls aged 8–18 years	subjects were classified as having reached puberty based on either hormone levels (total >50 ng/dL and free >5 pg/mL testosterone in boys and estradiol >20 pg/mL in girls) or onset of menarche	For boys, there was a relationship of reduced odds of reached puberty (raised testosterone) with increasing PFOS (delay of 190 days between the highest and lowest quartile) For girls, higher concentrations of PFOA or PFOS were associated with reduced odds of postmenarche (130 and 138 days of delay, respectively)	(Lopez-Espinosa et al., 2011) [243]
PFOS PFOA PFNA	2292 children (6–9 years of age)	estradiol, total testosterone, and IGF-1	In boys, PFOA concentrations were significantly associated with testosterone levels; PFOS with estradiol, testosterone, and IGF-1; and PFNA with IGF-1 In girls, significant associations were found between PFOS and testosterone and IGF-1; and PFNA and IGF-1	(Lopez-Espinosa et al., 2016) [244]
PFOS PFOA	424 mother-infant pairs	estrone (E1), b-estradiol (E2), and estriol (E3), infants: head circumference, body weight, body length	PFOS was positively related to E1 and E3, but negatively related to E2 Serum PFOA was positively related to serum E1 and negatively related to head circumference at birth Serum E2 was negatively related to head circumference, body weight, and body length at birth and serum E3 was positively related to body weight Serum E3 mediated the relationship between serum PFOS and body weight PFAS could affect estrogen homeostasis and fetal growth during pregnancy and estrogens might mediate the association between exposure to PFAS and fetal growth	(Wang et al., 2019) [245]
PFOS PFOA	47,092 adults	alanine transaminase (ALT), γ-glutamyltransferase (GGT), direct bilirubin	Positive association between PFOA and PFOS concentrations and serum ALT level, a marker of hepatocellular damage. The relationship with bilirubin appears to rise at low levels of PFOA and to fall again at higher levels.	(Gallo et al., 2012) [246]
PFHpA PFOA PFNA PFDA PFUnDA PFDoDA PFHxS PFOSA	1002 individuals from Sweden (50% women) at ages 70, 75 and 80	bilirubin and hepatic enzymes alanine aminotransferase (ALT), alkaline phosphatase (ALP), and γ-glutamyltransferase (GGT)	Positive associations of PFHpA, PFOA, PFNA, PFDA, and PFUnDA with ALP Concentrations of PFHpA, PFOA, PFNA, and PFOS were positively associated with the activity of ALT The changes in PFAS concentrations were positively associated with GGT and inversely associated with the changes in circulating bilirubin	(Salihovic et al., 2018) [30]
PFOS PFOA PFHxS	3297 participants from Ronneby, a municipality with drinking water highly contaminated by PFAS (exposed group)	thyroid hormone levels, with adjustments for age, sex and BMI	No associations between PFAS and thyroid hormones in adults and seniors except for a positive association between PFAS and fT4 in males over 50 Higher thyroid hormone levels in the preteen children from Ronneby compared to the reference group Weak evidence of associations between increased PFAS levels and decreased fT3 in preteen boys, and decreased TSH in teenage males	(Y. Li et al., 2021) [247]
PFOA PFOS	101 healthy 1-year-old children	Antibodies against haemophilus infuenza type b, tetanus and diphtheria, interferon gamma, cholesterol	Significant associations between PFOA, but not PFOS concentrations, and adjusted levels of vaccine antibodies against haemophilus influenza type b, tetanus and diphtheria PFOA levels inversely related to the interferon gamma (IFN) production of ex-vivo lymphocytes after stimulation with tetanus and diphtheria toxoid No infuence of PFOA and PFOS on infections and cholesterol level during the frst year of life	(Abraham et al., 2020) [248]
PFOA PFOS	1146 children	serum concentrations of specific IgG antibodies against tetanus and diphtheria at ages 5 and 7	Approximate BMDL of 1 ng/mL serum for both PFOS and PFOA for the serum concentrations of specific IgG antibodies against tetanus and diphtheria at ages 5 and 7 Proposed reference concentration of about 0.1 ng/mL as the serum-based target	(Budtz-Jørgensenet al., 2018) [249]
PFHxS, PFOS, PFOA, PFDA, PFNA	275 males and 349 females participated in clinical examinations and provided blood samples at ages 18 months and 5 years	serum concentrations of antibodies against tetanus and diphtheria vaccines determined at age 5	Pre-natal exposure showed inverse associations with the antibody concentrations five years later, with decreases by up to about 20% for each two-fold higher exposure Associations for serum concentrations at 18 months and 5 years were weaker Concentrations estimated for ages 3 and 6 months showed the strongest inverse associations with antibody concentrations at age 5 years, particularly for tetanus Joint analyses showed statistically significant decreases in tetanus antibody concentrations by 19–29% at age 5 for each doubling of the PFAS exposure in early infancy	(Grandjean et al., 2017) [250]
PFHxS, PFOA, PFOS, PFNA, PFDA.	516 subjects	PFAS serum concentrations and concentration of antibodies against diphtheria and tetanus	Diphtheria antibody concentrations decreased at elevated PFAS concentrations at 13 y and 7 y; the associations were statistically significant for perfluorodecanoate (PFDA) at 7 y and for perfluorooctanoate (PFOA) at 13 y, both suggesting a decrease by ∼25% for each doubling of exposure Structural equation models showed that a doubling in PFAS exposure at 7 y was associated with losses in diphtheria antibody concentrations at 13 y of 10–30% for the five PFAS	(Grandjean et al., 2017) [251]

## Data Availability

No new data were created or analyzed in this study. Data sharing is not applicable to this article.
